# Architecture of the native major royal jelly protein 1 oligomer

**DOI:** 10.1038/s41467-018-05619-1

**Published:** 2018-08-22

**Authors:** Wenli Tian, Min Li, Huiyuan Guo, Wenjun Peng, Xiaofeng Xue, Yifan Hu, Yang Liu, Yazhou Zhao, Xiaoming Fang, Kai Wang, Xiuting Li, Yufeng Tong, Michael A. Conlon, Wei Wu, Fazheng Ren, Zhongzhou Chen

**Affiliations:** 1 Beijing Advanced Innovation Center for Food Nutrition and Human Health, State Key Laboratory of Agrobiotechnology, China Agricultural University, Beijing, 100193 China; 2 Institute of Apicultural Research, Chinese Academy of Agricultural Sciences, Beijing, 100093 China; 3 Beijing Advanced Innovation Center for Food Nutrition and Human Health, Beijing Technology and Business University, Beijing, 100048 China; 4 Structural Genomics Consortium, University of Toronto, Toronto, Ontario M5G 1L7 Canada; 5 Department of Pharmacology and Toxicology, University of Toronto, Toronto, Ontario M5S 1A8 Canada; 6 CSIRO Health and Biosecurity, Adelaide, SA 5000 Australia

## Abstract

Honeybee caste development is nutritionally regulated by royal jelly (RJ). Major royal jelly protein 1 (MRJP1), the most abundant glycoprotein among soluble royal jelly proteins, plays pivotal roles in honeybee nutrition and larvae development, and exhibits broad pharmacological activities in humans. However, its structure has long remained unknown. Herein, we identify and report a 16-molecule architecture of native MRJP1 oligomer containing four MRJP1, four apisimin, and eight unanticipated 24-methylenecholesterol molecules at 2.65 Å resolution. MRJP1 has a unique six-bladed β-propeller fold with three disulfide bonds, and it interacts with apisimin mainly by hydrophobic interaction. Every four 24-methylenecholesterol molecules are packaged by two MRJP1 and two apisimin molecules. This assembly dimerizes to form an H-shaped MRJP1_4_-apisimin_4_-24-methylenecholesterol_8_ complex via apisimin in a conserved and pH-dependent fashion. Our findings offer a structural basis for understanding the pharmacological effects of MRJPs and 24-methylenecholesterol, and provide insights into their unique physiological roles in bees.

## Abstract (web summary)

Major royal jelly protein 1 (MRJP1) is the most abundant glycoprotein in royal jelly (RJ). Here the authors isolated MRJP1 from RJ and determined the 2.65 Å resolution crystal structure of the 16-molecule oligomer, which also contained 24-methylenecholesterol and apisimin bound to MRJP1.

## Introduction

Royal jelly (RJ) is mixture of natural products secreted from the hypopharyngeal and mandibular glands of nurse honeybees (*Apis mellifera*)^1^. RJ is essential for queen reproduction and larvae development in honeybee colonies, and is now widely used in human health and beauty products. Castes are nutritionally regulated, and only larvae exclusively fed on RJ develop into queens^2–4^. Intriguingly, queen honeybees have an approximately twofold larger body size and tenfold longer lifespan than worker bees of the same genotype.

RJ contains 12–15% protein, of which 82–90% are members of the major royal jelly protein family (MRJPs) that share high homology, and are designated MRJP1−9^5–7^. MRJPs have a common evolutionary origin with the Yellow protein family from insects and some bacteria^8^. MRJP1 is the most important and abundant glycoprotein in RJ and in bee bread, accounting for 48% of water-soluble RJ proteins^1^, and is also the most prominent colony-specific honeybee protein^9^. Besides their nutritional role, MRJP1 is a key factor responsible for honeybee development^10–12^. Furthermore, MRJP1 plays important roles in bee brain functions such as learning, memory, and social behaviour^9,13^. MRJP1 also exhibits a broad range of pharmacological activities in human health, such as promoting cell growth and wound healing^14–19^, broad-spectrum antibacterial and antifungal activities^20,21^, hypocholesterolemic effects^22^, antitumor activity^23^, vasodilative and anti-hypertension activity^24,25^, and immune enhancement^26,27^.

Soluble MRJP1 is present in both monomeric and oligomeric forms in RJ^1^. It exists predominantly as an oligomer in physiological conditions^2,28,29^. In particular, the MRJP1 oligomer (also called apalbumin) has significantly higher heat resistance and longer storage stability than the monomeric form^30^. This underscores the significance of the oligomer when studying the biological properties of MRJP1. Interestingly, the MRJP1 oligomer reportedly comprises both MRJP1 and apisimin. Apisimin is a serine-valine-rich peptide exclusively found in honeybee and has the tendency to form oligomers^31^. However, the composition, molecular weight, and binding stoichiometry of the MRJP1 oligomer remain controversial^16,28,31,32^. The molecular weight of the MRJP1 oligomer has been estimated to be 280 kDa^2^, 290 kDa^28^, 350 kDa^17,33^, 420 kDa^1^, or 450 kDa^10^. Moreover, no structures of the intriguing MRJP family members have been resolved at the atomic level^5^, which hampers rational understanding and drug applications.

Herein, we identify the components of the large MRJP1 oligomer, and determined the structure at 2.65 Å. 24-methylenecholesterol is unexpectedly found in it. To the best of our knowledge, no structures of MRJP family member proteins, apisimin, and 24-methylenecholesterol are reported previously. The overall architecture of MRJP1_4_-apisimin_4_-24-methylenecholesterol_8_ reveals a quaternary structure consisting of a dimer of dimers, in which eight 24-methylenecholesterol molecules are packaged. Moreover, our biochemical, biophysical, and crystal structure analyses demonstrate that the assembly states of MRJP1 oligomers are highly conserved and pH-dependent. Interaction between two apisimin proceeds via a mode consisting mostly of hydrophobic contacts. Taken together, our findings provide a structural basis for a better understanding of the unique physiological roles of MRJP family proteins and 24-methylenecholesterol in bees and their pharmacological effects in humans.

## Results

### Structure determination of the MRJP1 oligomer

Previous studies indicated that MRJP1 exhibits a relatively high degree of disorder^32^. Various constructs and fusion tags have been tested to express MRJP1 in *E. coli* and yeast systems. However, it was difficult to obtain sufficient amounts of soluble recombinant MRJP1 for X-ray crystallographic analysis. We therefore attempted to purify native MRJP1 from RJ. Using ultrafiltration, and ion-exchange and gel filtration chromatography steps, both the MRJP1 monomer and oligomer were purified to high homogeneity (Supplementary Fig. 1A, B). To further confirm the purified MRJP1 proteins, matrix-assisted laser desorption/ionization-time-of-flight (MALDI-TOF) mass spectrometry (MS) and liquid chromatography (LC–MS/MS) were performed. For the oligomer, molecular mass analysis by MALDI-TOF MS identified the digested peptides as MRJP1 following database searches, with 84% coverage, and LC–MS/MS spectra further confirmed the peptide sequences (Supplementary Fig. 1C−F). The identity of the MRJP1 monomer was similarly confirmed by MS.

During initial screening, only the native MRJP1 oligomer successfully yielded crystals (Supplementary Fig. 2A). But the small needle-like crystals did not grow large, and the diffraction quality was poor, even after extensive optimisation of the crystallisation conditions using additives, detergents, and seeding (Supplementary Fig. 2B). Therefore, several protein treatments, such as protease digestion, lysine methylation^34^, and deglycosylation were performed. Lysine methylation increased the size of crystals (Supplementary Fig. 2C), but the diffraction resolution remained low. Interestingly, the native MRJP1 oligomer was only partly deglycosylated by peptide:N-glycanase (PNGase) F under mild conditions (Supplementary Fig. 3A), and several crystal forms were obtained after deglycosylation of MRJP1 oligomers (Supplementary Fig. 2D−F). Fortunately, high-quality diffraction data were eventually obtained using cuboid crystals (Supplementary Fig. 4A, B).

**Fig. 1:**
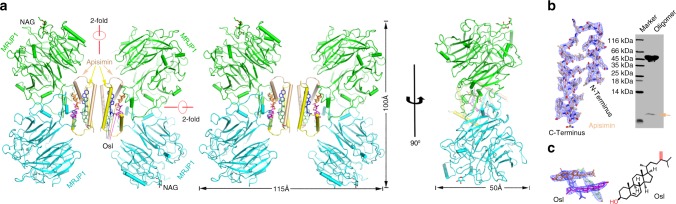
Structure of the MRJP1 oligomer. **a** The H-like complex structure contains four MRJP1 (green or cyan), four apisimin (yellow or wheat), and eight 24-methylenecholesterol (blue, purple, orange, or lime sticks) molecules. N-acetylglucosamine (NAG) attached to Asn144 is shown in stick representation. The structure has two orthogonal twofold axes. Note that the stereoview is shown in the left and middle panels, a 90° rotation view in the right panel. The composite simulated-annealing *mF*_*o*_—*DF*_*c*_ omit electron density maps of apisimin (**b**) and 24-methylenecholesterol (**c**) (also called Ostreasterol, Osl; formula shown in the right panel) are shown at 3.0 σ. The presence of apisimin (wheat arrow) was detected using tricine-SDS-PAGE

Because, the sequence homology between MRJP1 and any other protein of known three-dimensional structure was <17%, the structure were determined by combining multiple isomorphous replacement and molecular replacement methods, and refined to a resolution of 2.65 Å in space group of *H*32, with an *R*_work_ of 23.9% and an *R*_free_ of 27.5% (Table 1). The MRJP1 molecule possesses two orthogonal twofold axes, as confirmed by self-rotation analysis (Fig. 1a and Supplementary Fig. 4C).

**Table 1:** Data collection and refinement statistics of MRJP1-apisimin-24-methylenecholesterol complex

	Pt-soaked	Br-soaked	I-soaked	Native
*Data collection*				
Wavelength (Å)	0.9789	0.9201	1.5400	0.9793
Space group	*H*32	*H*32	*H*32	*H*32
Cell dimensions				
*a*, *b*, *c* (Å)	208.9, 208.9, 146.6	211.0, 211.0, 150.0	208.8, 208.8, 146.6	211.6, 211.6, 150.0
*α*, *β*, *γ* (°)	90, 90, 120	90, 90, 120	90, 90, 120	90, 90, 120
Resolution (Å)	50.0–3.00 (3.05–3.00)	50.0–3.11 (3.17–3.11)	50.0–4.00 (4.08–4.00)	50.0–2.65 (2.7–2.65)^a^
*R*_sym_ (%)	9.1 (76.3)	10.2 (62.3)	9.9 (43.4)	9.9 (301.9)
*R*_meas_ (%)				10.6 (322.2)
*R*_pim_ (%)				3.7 (111.8)
CC_1/2_				1.00 (0.31)
Wilson B factor (Å^2^)				49.4
*I*/σ*I*	13.1 (1.4)	16.0 (1.8)	6.5 (1.6)	20.5 (0.81)
Completeness (%)	98.7 (99.6)	91.9 (92.4)	93.4 (85.7)	99.9 (100.0)
Total number of reflections	1,120,113	980,823	580,516	945,275
Unique reflections	24,643	24,430	12,222	37,682
Redundancy	4.3 (4.3)	6.9 (6.9)	3.1 (2.7)	8.2 (8.1)
Refinement				
Resolution (Å)				50–2.65 (2.72–2.65)
Unique reflections				37682 (289)
* R*_work_/*R*_free_ (%)				23.9/27.5 (34.5/45.8)
* Number of atoms*				
Protein				6303
Ligands				116
Water				144
*B-factors (Å* ^*2*^ *)*				
Protein				49.22
Ligands				43.18
Water				35.57
*RMS deviations*				
Bond lengths (Å)				0.010
Bond angles (°)				1.46
Ramachandran Plot (%)^b^				83.6/15.9/0.5/0.0

*R*_sym_ = ∑_*h*_∑_*i*_*|I*_*h,i*_*-I*_*h*_*|/*∑_*h*_∑_*i*_*I*_*h,i*,_ where *I*_*h*_ is the mean intensity of the *i* observations of symmetry related reflections of *h*

^a^Statistics for highest resolution shell

^b^Residues in most favored, additional allowed, generously allowed, and disallowed regions of the Ramachandran plot

### Apisimin-dependent dimerisation of dimers MRJP1 structure

The asymmetric unit contains two MRJP1, two apisimin, four unexpected 24-methylenecholesterol (ostreasterol, Osl), and 144 water molecules. The electron density maps clearly showed electron densities for bound apisimin (Supplementary Fig. 5B). Moreover, the composite simulated-annealing *mF*_*o*_*—DF*_*c*_ omit electron density map further confirmed the presence of apisimin (Fig. 1b). For both MRJP1 and apisimin, mature proteins were detected without N-terminal signal peptides. For MRJP1 molecules, protomer A spans residues 23–432 and protomer B spans residues 28–432 with N-acetylglucosamine linked to Asn144. Residues 197–199 and 290–291 in protomer A, residues 117, 120, 172–173, 183, 192–194, 196−199, 219–220, 242, 245–247, 284−288, 291−294, and 389–391 in protomer B could not be modelled due to disorder (Supplementary Fig. 6A), which may indicate conformational flexibility of these residues. For two apisimin molecules, protomer C spans residues 37–77 and protomer D spans residues 36–78. The two protomers of MRJP1 and apisimin are almost identical, with an overall root-mean-square deviation (RMSD) for all C_α_ atoms of 0.78 Å and 0.93 Å, respectively (Supplementary Fig. 6A, B). Details of the data collection and processing statistics are shown in Table 1.

The complete MRJP1-apisimin-24-methylenecholesterol ternary structure is obtained by applying a twofold symmetry operation. The overall complex adopts an H-shaped structure with approximate height, width, and length of 100 × 50 × 115 Å, respectively. It has an apisimin-dependent dimerisation of MRJP1 dimers quaternary structure, comprising 16 molecules, including four MRJP1, four apisimin, and eight 24-methylenecholesterol molecules (Supplementary movie 1).

### The MRJP1 oligomer contains 24-methylenecholesterol

During refinement of the MRJP1 oligomer structure, we noticed four large electron density features located between two apisimin molecules in the asymmetric unit (Supplementary Fig. 5B), in which Osl fitted well. Moreover, the composite simulated-annealing *mF*_*o*_*—DF*_*c*_ omit electron density map further confirmed the presence of Osl (Fig. 1c). Osl was previously reported to be present in RJ and honeybees^35,36^. Thus, to exclude any possible contamination from RJ directly, we first purified the MRJP1 oligomer to high homogeneity by ultrafiltration, ion-exchange and gel filtration chromatography steps. The unknown small molecule was then released from the purified MRJP1 oligomer by adding 2M NaOH and extracted with n-hexane. Three different methods were used to identify the small molecule, namely electron ionization gas chromatography–mass spectrometry (EI-GC/MS), ^1^H, and ^13^C NMR (Supplementary Fig. 7), and the results matched perfectly with those of the Osl standard/ reference compound^37^, confirming its identity.

Due to the existence of non-crystallographic twofold symmetry, the positions of the four Osl molecules could be divided into inner and outer types (Supplementary Fig. 6E). Each of the inner Osl molecules contact two MRJP1, one apisimin, and the other three Osl molecules in the asymmetric unit (Supplementary Fig. 6F), while each of the outer Osl molecules only interact with two apisimin molecules and the other three Osl molecules.

### Disulfide-bond stabilised six-bladed β-propeller fold

**Fig. 2:**
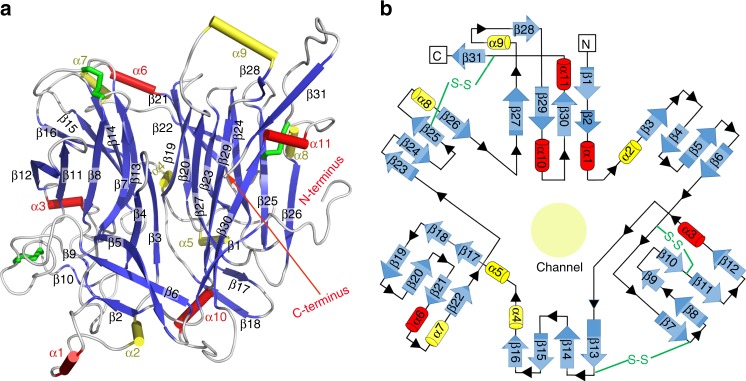
Structural characterisation of the MRJP1 protomer. Secondary structures (**a**) and topology (**b**) of MRJP1 showing a six-bladed β-propeller fold. Three disulfide bonds and the central channel are shown

**Fig. 3:**
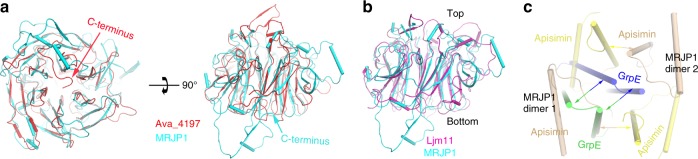
Overlay of MRJP1 and apisimin with their closest structural homologs. Overlay of MRJP1 with Ava_4197 (PDB: 2qe8, **a**) and Ljm11 (PDB: 3q6k, **b**). **c** Overlay of the apisimin tetramer with the closest structural homolog, GrpE (PDB: 3a6m)^40^. Interactions between protomers are indicated by coloured arrows

MRJP1 is folded into a unique six-bladed β-propeller shape containing 31 β-strands and 11 α-helices with a central channel (Fig. 2). The protein fold is similar to that of LJM11 from sand fly *Lutzomyia longipalpiss*^38^, the template structure used for molecular replacement (Fig. 3b). Each blade consists of a four-stranded β-sheet, and the fourth β-strand of each blade connects with the first β-strand of the subsequent one to form a bucket. The wall of the central channel consists of a parallel arrangement of the first β-strands from each blade (Fig. 2). A series of large loops, β-strands, and α-helices are in the bucket periphery. The temperature factors of these peripheral regions are high, suggesting they are flexible (Supplementary Fig. 6C). A DALI search^39^ of the MRJP1 structure identified an uncharacterised protein, Ava_4197 from *Anabaena variabilis* (PDB ID 2qe8, RMSD = 2.4 Å, *Z* = 34.6) as the closest structural homolog. Structural alignment between MRJP1, Ava_4197, and LJM11 indicated that the position of the core six-bladed β-propeller was similar, while the peripheral bucket was somewhat more variable in terms of the number and position of α-helices, β-strands, and loops (Fig. 3a, b). Interestingly, the C-terminus of MRJP1, which interacts with a neighbouring MRJP1, is much longer than those of Ava_4197 and LJM11 (Fig. 3a, b, and Supplementary Fig. 8A). Moreover, very low sequence conservation is observed between MRJP1 and other Yellow protein family members (Supplementary Fig. 8A), suggesting MRJP1 might function differently from other Yellow proteins.

Three disulfide bonds (Cys132–Cys195, Cys118–Cys150, and Cys329–Cys416) were observed in MRJP1, with the last one stabilising the C-terminal portion (Supplementary Fig. 6A). All cysteine residues in the last two disulfide bonds of MRJP1 are conserved in homologous members of the MRJP family (Supplementary Fig. 8B), suggesting that the overall structure might be conserved, and both disulfide bonds are essential for structural stability. By contrast, the Cys132–Cys195 disulfide between two blades of MRJP1 is not conserved in other MRJPs, and the most substantial differences are observed in the C-terminal region (Supplementary Fig. 8B). In addition to the very low sequence homology between MRJP1 and other Yellow family members, the Cys118–Cys150 disulfide bond is not present in LJM11^38^, and no disulfide bond was found in Ava_4197, highlighting the divergence of MRJPs from other Yellow protein family members. Overall, the marked differences in disulfide bonds and other sequence features may indicate that MRJP1 is functionally different from its homologs.

### The apisimin structure and interaction mode

All residues in the determined apisimin structure are conserved across different species (Supplementary Fig. 8C), indicating that the overall structure may be similar. Each apisimin molecule in the asymmetric unit contains two α-helices connected by a loop, with two hydrogen bonds and a large hydrophobic zone between the two α-helices, giving apisimin a relatively stable 47° oblique structure (Supplementary Fig. 6B). Consistently, both termini appear to be more flexible than the central region, as reflected by their higher temperature factors (Supplementary Fig. 6D).

A DALI search of the apisimin structure identified the chaperone GrpE (PDB 3a6m, RMSD = 4.2 Å, *Z* = 5.3)^40^ as the closest structural homolog (Fig. 3c). However, the sequence similarity is <10%, indicating low homology. Additionally, only interaction between apisimin protomers from different MRJP1 dimers is observed, and apisimin molecules in the same MRJP1 dimer do not interact. By contrast, in GrpE, interactions occur between two protomers from the same GrpE dimer (Fig. 3c). Furthermore, only the C-terminal α-helix of apisimin is involved in interactions, and hence forming the MRJP1 oligomer (MRJP1_4_-apisimin_4_-Osl_8_), which is clearly distinct from GrpE in which both helices are involved (Fig. 3c). Moreover, the interface area of each apisimin is only 509 Å^2^, which is significantly lower than the 3051 Å^2^ for each GrpE molecule, as calculated by the PISA server^41^. Taken together, these findings suggest that the spatial arrangement, interaction mode, and oligomeric packing of apisimin are markedly different from the closest structural homolog. Therefore, the apisimin structure reveals an unusual interaction mode.

### Extensive hydrophobic interactions are crucial for oligomer

**Fig. 4:**
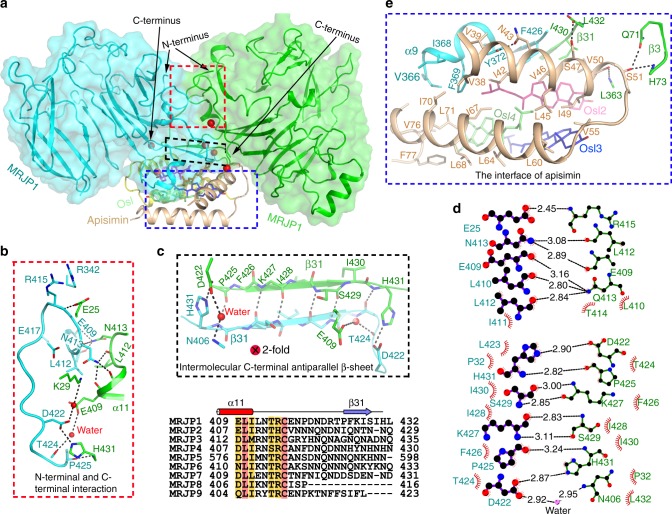
Detailed interactions of MRJP1 and apisimin in the MRJP1 dimer. **a** Overview of the three protein interfaces. Details of the symmetric intermolecular C-terminal and N-terminal interactions (**b**) and intermolecular C-terminal antiparallel β-sheets β31 (**c**), and their interactions (**d**) between MRJP1 protomers were calculated by Ligplot^70^. Hemispheres represent hydrophobic interactions, and lines represent polar interactions. Residues from the two protomers are shown on the left and right, respectively. All residues involved in the hydrophobic interactions are shown in black. For hydrophilic interactions, residues are shown in red or blue. **e** Details of the interactions of apisimin

**Fig. 5:**
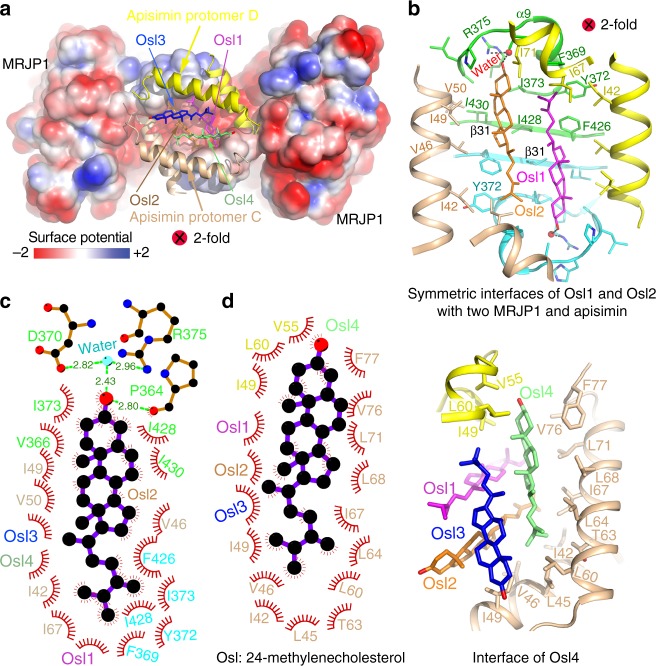
Detailed interactions of Osl in the MRJP1 oligomer. **a** Four Osl molecules in the surface potential of MRJP1. **b** Interactions of the inner two Osl molecules with MRJP1 and apisimin. **c** Interactions of Osl2 calculated by Ligplot. **d** Interactions between the outer Osl4 and surrounding residues

In the asymmetric unit (MRJP1_2_-apisimin_2_-Osl_4_), there exist two interfaces between MRJP1 protomers; symmetric intermolecular C-terminal and N-terminal interactions, and interactions between intermolecular antiparallel C-terminal β-sheets β31 (Fig. 4a–d). These interactions are strong, with a buried interface area of 771 Å^2^, and include extensive hydrophobic interactions, six salt bridges, and 16 hydrogen bonds (Fig. 4b–d). Most of the interacting residues are positioned at both termini, including Glu25, Lys29, Pro32, Arg342, Glu409, Leu410, Ile411, Asn413, Thr414, Arg415, Asp422, Thr424, Phe426, Ile428, Ser429, Ile430, and His431. Moreover, the temperature factors of the residues in the C-terminal β31 are 42% lower than the mean value, suggesting β31 is involved in forming a tight complex (Supplementary Fig. 6C). Additionally, two apisimin and the inner pair of Osl molecules also greatly contribute to the binding interface (Figs. 4e, 5b), resulting in a total buried interface of 1760 Å^2^ for each MRJP1, which strongly stabilises the dimer.

For apisimin and Osl molecules in the MRJP1 dimer, most of the interactions are also hydrophobic. Apisimin interacts with two MRJP1 and three Osl molecules with a total buried interface area of 1168 Å^2^ (Fig. 4e). For the inner two Osl molecules, each is totally enveloped by two MRJP1 molecules, one apisimin, and the other three Osl molecules (Fig. 5a–c and Supplementary Fig. 6F). For the outer two Osl molecules, each interacts with two apisimin molecules and the other three Osl molecules (Fig. 5d), with 92% of the total buried surface involved in the interaction. Meanwhile, no interactions occur between MRJP1 and the outer two Osl molecules. Interestingly, the inner and outer Osl molecules are almost parallel to the N-terminal or C-terminal α-helices of apisimin, respectively, ensuring maximal interaction. Taken together, these strong interactions render the entire MRJP1 dimer highly stable.

### MRJP1 oligomer is mainly tetrameric and is pH-dependent

**Fig. 6:**
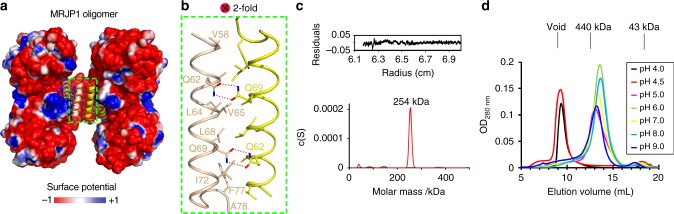
Structural basis of the formation of the MRJP1 oligomer. **a** The surface potential (±1 kBT/e) of the MRJP1 oligomer. The surface coloured as a gradient ranging from red (negative) to blue (positive). Details of the interfaces between apisimin and the neighbouring symmetry-generated apisimin molecule are shown in **b**. Two apisimin protomers are related by a twofold rotation axis, perpendicular to the plane of the page. **c** Analytical ultracentrifugation of the MRJP1 oligomer at pH 8. The c(S) distribution from sedimentation velocity analysis is shown. **d** Gel filtration of the MRJP1 oligomer at different pH values

Careful structural analysis revealed two symmetric interfaces between two apisimin molecules from two neighbouring MRJP1 dimers. Each interface is formed mainly by hydrophobic interactions and four additional hydrogen bonds (Fig. 6a, b and Supplementary Fig. 6G). Interestingly, only the C-terminal α-helices of the four apisimin molecules are involved in forming the interfaces. The residues involved in interface formation are conserved across different species (Supplementary Fig. 8C), revealing the importance of apisimin in the MRJP1 oligomer. The total buried interface area reaches 1117 Å^2^, slightly less than the value of 1600 ± 400 Å^2^ that is generally believed to be of physiological significance^42^. Furthermore, the free energy of assembly/association (ΔG^ass^) of the whole-MRJP1 oligomer was −9.3 kcal/mol, as calculated by the PISA server^41^. A large, negative value of ΔG^ass^ is indicative of a strong interface, suggesting the interface between the two apisimin molecules in the two MRJP1 dimers is sufficient for tetramer formation. Taken together, the evidence suggests the complete MRJP1 oligomer (MRJP1_4_-apisimin_4_-Osl_8_) is formed by apisimin-dependent dimerisation of MRJP1 dimers, and the four apisimin molecules are the key bridges holding the complex together.

To further investigate the oligomeric states of MRJP1 oligomers in solution, we performed gel filtration, sedimentation velocity experiments, and small-angle X-ray scattering (SAXS) analyses. The molecular weight of the native MRJP1 oligomer was determined by analytical ultracentrifugation to be 254 kDa (Fig. 6c). MRJP1 monomer, dimer, and oligomer were observed in analytical ultracentrifugation. Gel filtration and SAXS analyses further confirmed that the MRJP1 oligomer mainly exists as a MRJP1 tetramer at near-neutral pH (Supplementary Figs. 10C, 9). Therefore, the solved structure was likely that of the predominant oligomeric state in solution, and not an artefact of crystal packing. Moreover, gel filtration chromatography showed that the MRJP1 tetramer existed universally in RJ collected from the nectar of different plants and honeybee species (Supplementary Fig. 10A, B).

Interestingly, the MRJP1 oligomer has been reported to adopt different assembly states and molecular weights, ranging from 231 to 450 kDa, and large soluble aggregates have also been reported^1,2,10,17,28,32,33^. Upon reviewing the literature, we noticed that these analyses were carried out under different conditions. We think that pH might be the main cause. To evaluate the influence of pH on the oligomeric state of MRJP1, we performed gel filtration experiments on the purified MRJP1 oligomer using a Superdex-200 HR10/300 column (GE Healthcare) at a variety of different pH values (Fig. 6d). Between pH 5 and pH 9, the peak of the MRJP1 oligomer eluted at 13.6 mL, corresponding to the molecular weight of the MRJP1_4_-apisimin_4_-Osl_8_ complex. However, at pH 4.5 and pH 4, the peak of higher oligomer eluted at 9.9 mL, close to the void volume of the column, indicating the presence of larger soluble aggregates. To eliminate the possibility that MRJP1 might behave differently depending on treatment, we purified MRJP1 from RJ in buffer at a different pH, and the results were comparable to the original pH (Supplementary Fig. 10C). By contrast, the behaviour of the MRJP1 monomer did not change at different pH. Taken together, these results suggest large soluble aggregates and the MRJP1_4_-apisimin_4_-Osl_8_ complex are interchangeable, consistent with previous observations^3,43^.

## Discussion

The honeybee is one of the most familiar and important insects for humans and plants, critical for apiculture and pollination, and its benefits are arguably immeasurable. RJ are natural products that are vital for honeybee development and reproduction, and widely used in human health-promoting foods, and cosmetics. The MRJP1 monomer (royalactin) rather than the oligomer is reported to be the key factor inducing queen differentiation in honeybees^10,12^. This raises the question why the more abundant MRJP1 oligomer does not have this function. Our structural analyses of the MRJP1 oligomer show that 10% surface of MRJP1 is involved in binding apisimin and Osl, especially in the C-terminal region. We suspect that the binding of apisimin and Osl in the MRJP1 oligomer block driving queen development through the downstream signalling pathways.

Over the years, the honeybee MRJP1 oligomer has been reported to be a hexamer, pentamer^28^, or tetramer^32^, with varying molecular mass^43^. Moreover, it is unclear which factors influence the oligomerisation of MRJP1, and whether the oligomeric state varies with the source of RJ. In this work, the native MRJP1 oligomer was purified from RJ and determined by analytical ultracentrifugation as a 254 kDa assembly at neutral pH. The structure of the MRJP1 oligomer was determined at the same pH as the insect midgut, and therefore likely represents the physiologically relevant and native structure. MRJP1 oligomer mainly exists as a MRJP1_4_-apisimin_4_-Osl_8_ complex and adopts an elegant H-like shape at near-neutral pH, and this was further confirmed by SAXS analysis (Fig. 6c and Supplementary Fig. 9). Interestingly, although the MRJP1 tetramer is the most common state, monomers and dimers were also observed. To investigate why, we performed a series of structural analyses. Apisimin functions as a dimer joining the protein complex, stabilising the complete MRJP1_4_-apisimin_4_-Osl_8_ tetramer. The tetramer interface area of 1117 Å^2^ is lower than the total dimeric interface area of 1760 Å^2^, and slightly less than the lower limit of 1600 ± 400 Å^2^ that is the assumed cutoff for physiologically relevant interfaces. Therefore, the MRJP1 tetramer is not highly stable, and may disassociate into dimers or even monomers to some extent, which could contribute to its in vivo function in honeybees.

We detected two types of MRJP1 oligomer in solution, and self-assembly was pH-dependent and reversible, as confirmed by size exclusion chromatography and MS. The MRJP1 tetramer was completely converted to large aggregates when the pH was lower than 4.5, close to the pH of natural RJ. Moreover, this phenomenon is universally conserved among all different sources of MRJP1, including various nectar plants and honeybee species (Supplementary Fig. 10A, B). Further structural analyses suggest that nine histidine residues (with pK_2_ = 6.0) on the protein surface may play crucial roles in the pH-dependent self-association of the MRJP1 oligomer. Our results of pH-dependent self-assembly size are similar to the recent online publication^11^. The larger soluble aggregates of MRJP1 oligomer form long fibrous structures that build the basis for the high viscosity of RJ to hold queen larvae on the RJ surface and prevent them falling out of their cells.

Structural analyses revealed that both termini of MRJP1 play key roles in forming the MRJP1 dimer (Fig. 4a−d). Indeed, the C-terminus of MRJP1 interacts with the other MRJP1 of the dimer, as well as with apisimin and Osl, thereby determining the formation of the MRJP1 dimer. Sequence alignment revealed that both termini and the regions involved in forming the MRJP1 dimer are highly diverse across the MRJP family (Supplementary Fig. 8B), which may explain why oligomers were not found for other MRJPs, with the exception of MRJP3, which forms a trimer^44^.

The surface of the MRJP1 oligomer is highly negatively charged at near-neutral pH (Figs. 5a, 6a), presumably resulting in repulsion between oligomers, making them resistant to aggregation under near-neutral conditions, and thus highly stable. Moreover, extensive interactions are involved in forming the MRJP1 oligomer, and many surfaces are protected from proteases by other MRJP1, apisimin, and Osl molecules. Thus, the MRJP1 oligomer appears to be heat-stable and more resistant to degradation than monomer during storage^30,45^. Indeed, our synchrotron radiation circular dichroism experiments confirmed that the denaturation temperature of the MRJP1 oligomer was much higher than that of the monomer (Tm = 55 °C vs. 43 °C, Supplementary Fig. 11), consistent with previously reported observations^30,45^. Therefore, the MRJP1 oligomer is more stable than the MRJP1 monomer. Since other MRJPs appear not to form oligomers, their stability may also be much lower than that of the MRJP1 oligomer, which may explain why the MRJP1 oligomer is the major storage protein in RJ.

The honeybee provides a convenient classical model for understanding the biology of insects, epigenetics, and social systems. Diploid eggs become queens or workers depending on whether larvae are fed royal jelly (RJ) or not^4,46^. The role of RJ for caste determination in the honeybee is the textbook classic paradigm for environmental caste differentiation in social insects. The association between RJ and caste formation has been known for >100 years, but the identity of the component(s) in RJ that induce queen development has remained elusive and controversial^3,10,12^. It is believed that MRJP1 play pivotal roles in honeybee development and reproduction^11^. Our current findings suggest that large soluble aggregates of the MRJP1 oligomer in RJ might provide a means for storing the physiologically active MRJP1 in the colony. The pH of the insect midgut is normally above 5.5. After ingestion by bee larvae, the large soluble aggregates of the MRJP1 oligomer would be converted to the tetramer or even the monomer in the midgut. Interestingly, proteomic analyses revealed that queen development was not triggered by intact MRJP1 oligomer complexes, but rather by proteolytic products generated in the digestive tract^47^. Moreover, because the amino acid sequence of apisimin has several protease cleavage sites, including those recognised by chymotrypsin and trypsin, it also provides an opportunity to release MRJP1 from the MRJP1 oligomer in the honeybee digestive system. Moreover, apisimin can be separated from the MRJP1 oligomer under mild conditions^28^, implying easy conversion between oligomeric and monomeric MRJP1. Thus, the MRJP1 oligomer might be digested at somewhat lower rate than the monomer in the digestive tract, thereby fulfilling some biological functions.

Sand fly LJM11 confers protective immunity against *Leishmania* infection due to its high affinity for serotonin^38^. Since both MRJP1 and LJM11 belong to the same Yellow protein family, we investigated whether the MRJP1 oligomer and/or monomer could bind serotonin. Isothermal titration calorimetry (ITC) results showed that neither form could bind serotonin, at least not under the experimental conditions employed (Supplementary Fig. 12A, B). Structural alignment of the MRJP1 and Ljm11 proteins revealed that the central hole in MRJP1 is too small, and residues near the hole in MRJP1 would sterically clash with serotonin (Supplementary Fig. 12C−E). Moreover, we failed to find LJM11 when searching for homologs of the MRJP1 structure using the DALI server in protein data bank, revealing the low structural similarity between MRJP1 and LJM11. Therefore, unlike LJM11, MRJP1 cannot bind serotonin, implying a different function for MRJP1.

MRJP1 is the most abundant glycoprotein among the soluble proteins in RJ, and has three predicted glycosylation sites at Asn28, Asn144, and Asn177^48^. Our experiments clearly showed that N-linked glycan sites hindered the formation of tight crystal contacts. Interestingly, electron density for N-acetylglucosamine (NAG) was observed at glycosylation site Asn144 in the crystal structure (Supplementary Fig. 3). Although PNGase F was effective for deglycosylating N-linked glycans, NAG interacts with surrounding residues including Asn84, Val143, and Gln147, hindering its removal by PNGase F under mild conditions. Observation and structural analysis confirmed partial deglycosylation of the MRJP1 oligomer under native conditions (Supplementary Fig. 3A). Our structure therefore directly confirmed that native MRJP1 is glycosylated. Hypertension is a major risk factor that leads to cerebral stroke, heart failure, and acute myocardial infarction in humans. MRJP1 is a potential candidate for anti-hypertension, but interestingly, only glycosylated MRJP1 significantly inhibits the migration of human vascular smooth muscle cells (VSMCs), and therefore possesses anti-hypertension activity, unlike its deglycosylated form^24^, revealing the importance of glycosylation.

Osl is a natural product first identified in marine organisms, which is also found in RJ and honeybees^35,36^. In the present study, electron density, EI-GC/MS, and ^1^H and ^13^C NMR results confirmed its presence in the MRJP1 oligomer (Fig. 1c and Supplementary Fig. 7). To our knowledge, Osl has not been reported to exist in a protein complex. Although unexpected, Osl is a tightly bound cofactor in the MRJP1 oligomer. Rearing experiments reveal that Osl might greatly promote the growth of honeybee larvae. Importantly, Osl is known to significantly decrease cholesterol levels in both serum and liver^49^, and the MRJP1 oligomer exhibits high hypocholesterolemic activity in rats, although the mechanism remains to be elucidated^22^. Our discovery of Osl in the MRJP1 oligomer may explain its hypocholesterolemic effect, and provides a potential new strategy for lowering cholesterol in humans. Moreover, Osl is reported to have anti-aging effects and neuroprotective functions^50^, suggesting the MRJP1 oligomer may be used to develop promising neuroprotective drugs. Osl, constituting 49–58% of RJ sterols, also has a high-level content in the queens, but very little in worker honeybees or pollens^51^. Osl is an essential sterol in bee metabolism influencing moulting and the development of ovaries^52^. Moreover, it shows the strongest estrogenic activity among the main small compounds in RJ^35^. Therefore MRJP1 might play important roles in queen development, and have the potential to improve menopausal symptoms and bone formation, and prevent osteoporosis in humans.

We believe our findings will contribute to a better understanding of the unique pharmacological effects of MRJP1 in humans, and they provide insights into the mechanisms underlying its physiological functions in bees, including caste differentiation, learning ability, colony organisation, and development. Importantly, MRJPs have great potential as drug candidates for promoting human health, due to their hypocholesterolemic, anti-hypertension, antibiotic, anti-tumour, and cell growth-promoting activities, and their ability to prolong longevity and protect the immune system. For example, the MRJP1 glycoprotein harbours three antimicrobial jelleine peptides^53^.

## Methods

### Protein preparation

Fresh royal jelly (RJ) samples were collected from *Apis mellifera* and *Apis cerana* colonies located in several regions of Zhejiang province during the flowering periods of different nectar plants. All RJ samples were stored at −20 °C until analyses.

One gram of RJ was dissolved in 10 mL of 40 mM phosphate buffer (pH 8.0). The suspension was centrifuged (25,000 g) for 15 min at 4 °C to separate the supernatant and pellet, and the supernatant was filtered with the 0.45 μm membrane filter. After adding 30 mL 20 mM Tris-HCl (pH 8.0), the filtered fluid was concentrated to 5 mL by ultrafiltration through a 30 kDa MW cutoff membrane (Millipore). The concentrated solution was further purified by the Q sepharose (Bio-rad). The protein was eluted with a linear gradient of NaCl from 0 to 1.0 M at a flow rate of 0.5 mL/min, and the elution was detected by SDS-PAGE and Coomassie blue staining. The fractions containing MRJP1 were collected and concentrated to 2 mL. MRJP1 collected from Q sepharose was applied to a Hiload Superdex-200 26/600 column (GE Healthcare) previously equilibrated with the buffer containing 20 mM Tris-HCl (pH 8.0) and 150 mM NaCl. The column was then eluted at a flow rate of 1.5 mL/min, and the fractions containing MRJP1 monomer and oligomer protein were collected, respectively.

### Protein deglycosylation

N-linked deglycosylation was conducted with PNGase F (peptide:N-glycosidase F). PNGase F was added to MRJP1 protein and incubated overnight at 4 °C. Then the mixture was changed to buffer containing 20 mM Tris-HCl (pH 8.0) and 20 mM NaCl, then purified using UNO^TM^ Q (Bio-rad). Fractions containing MRJP1 were collected and concentrated to 15 mg/mL for crystallization.

### MALDI-TOF-MS of MRJP1 oligomer

The time-of-flight instrument (Autoflex III TOF/TOF, Bruker Daltonics) fitted with a 337-nm nitrogen laser in linear mode was used for peptide molecular weight determination. Peptides were analyzed in reflector mode with delayed extraction using an acceleration voltage of 20 kV. Data were generally collected after ionization at 100 MHz in the *m/z* 500 to 4000 range with the instrument calibrated externally using a mixture of human angiotensin II, ACTH clip (residues 18–39) and bovine insulin.

The purified MRJP1 oligomer was desalted by ultrafiltration using a 30 kDa MW cutoff membrane Amicon Ultra-15 centrifugal filter units (Millipore) to 2 mg/mL. Then the protein was digested by trypsin overnight. A total of 1 μL of the digested peptide was spotted and dried naturally and 2 μL of 5 mg/mL MALDI matrix α-cyano-4-hydroxycinnamic acid in 50% acetonitrile/0.1% TFA was spotted on the sample spot. The solvent evaporated and the peptide/matrix co-crystallized.

### Liquid chromatography/electrospray mass spectrometry

The HPLC Series 1100 system used for the analysis of the digested peptides was composed of a binary pump, a column temperature stabilizer and a diode array detector (Agilent, Waldbronn, Germany). Agilent ChemStation software was used for instrument control and data processing. A reversed-phase HPLC column (Zorbax 300SB-C18 150 × 2.1 mm, 5 μm) was used for peptide separation with the mobile phase flow rate set at 0.2 mL/min (solution A: 0.1% TFA in H_2_O (V/V); solution B: 0.1% TFA in acetonitrile (V/V)). For the separation, the solutions A and B were used as the gradient elution. The gradient program was set as follows: 5% B (0 min), 5% B (5 min), 40% B (40 min), 75% B (70 min), 75% B (80 min), 5% B (85 min) for total runtime of 85 min.

The mass spectra were obtained using a Finnigan LCQ ion trap mass spectrometer (Thermo electron, San Jose, CA, USA) equipped with an electrospray ionization source (Spectronex AG, Basel, Switzerland). The spray voltage and heated capillary were 4.5 kV and 275 °C, respectively. The scan range was set from m/z 400 to 2000. After electrospray ionization, tandem MS (MS/MS) data were obtained by collisional activating of the solute molecules colliding with argon gas. The collision energy value was set at 35%.

### Expression and purification of recombinant MRJP1

The cDNA encoding the mature protein, excluding the signal peptide, of full-length *MRJP1* (58–1296 bases, Genbank^TM^ accession number D79207) and various fragments were subcloned into pET19b (Merck), pGEX-4T-2 (GE), pCold™ TF (TaKaRa) or some other vectors using the primers in Supplementary Table 1. The method was similar to the reference^10^. Briefly, *Escherichia coli* strain BL21 (DE3) pLysS cells (Merck) harboring the plasmids were grown at 37 °C to an OD_600_ of ~0.8 in LB medium containing ampicillin (50 μg/mL). Then isopropyl β-D-thiogalactoside (0.5 mM) was added to induce protein expression, and culture was continued at 37 °C for 3 h or at 18 °C for 17 h. Then, the cells were collected by centrifugation, suspended in buffer A (50 mM Tris-HCl pH 8.0 containing 500 mM NaCl), and disrupted with a Tomy Ultrasonic Disruptor at 20 kHz and 80 W for 80 s. The sonicated solution was centrifuged at 20,000×*g* for 30 min at 4 °C, and the supernatant was applied to a His-Bind or GST resin affinity column and eluted with 400 mM imidazole or 10 mM glutathione, respectively. The Tag was removed by the corresponding protease at a 1:10 (w/w, protease/protein) ratio for 6 h at 4 °C to remove His, GST or TF Tag. The fractions containing MRJP1 were collected and applied to a HiPrep^TM^ 16/60 Sephacryl S-200 column. The column was eluted with Buffer A at a flow rate of 1.0 mL/min, and the fractions containing MRJP1 were pooled, dialyzed against distilled water and lyophilized. All the protein purification procedures were performed at 4 °C.

For the recombinant expression of MRJP1 in yeast, the method was according to the publication^54^.

### Lysine methylation

The method used is slightly modified from the publication^34^. The buffer was changed to 50 mM HEPES (pH 7.5), 250 mM NaCl at protein concentrations of 1 mg/mL. A total of 20 μl freshly prepared 1 M dimethylamine-borane complex (ABC) and 40 μl 1 M formaldehyde were added per mL protein solution, and the reactions were gently incubated at 4 °C for 2 h. A further 20 μl ABC and 40 μl formaldehyde per mL protein solution were added and the incubation continued for 2 h. Then a final addition of 10 μl ABC and 20 μl formaldehyde per mL protein solution, the reaction was incubated overnight at 4 °C. The protein was further purified by size exclusion chromatography columns in buffer A.

### Analytical size exclusion chromatography

The MRJP1 proteins were applied to a Superdex-200 10/300 column (GE Healthcare) equilibrated with a buffer containing 150 mM NaCl at 20 mM Tris (pH 7.0, 8.0, 9.0) or 20 mM sodium citrate (pH 4.0, 4.5, 5.0, 6.0). To compare the different states between MRJP1 oligomer and monomer, protein was exchanged to the corresponding buffer before loaded onto the Superdex-200 column. The proteins were detected by SDS-PAGE followed by Coomassie blue staining.

### Crystallization

Extensive crystallization screens were performed at both 18 °C and 4 °C for native, lysine methylated and deglycosylated MRJP1 with concentration at 20–25 mg/ml. Monomer MRJP1 failed to grow a crystal after many trials. For native MRJP1 oligomer crystal screen, only needle clusters were obtained in several crystal conditions and the extensive optimization could not change the crystal shape. Lysine methylated MRJP1 oligomer were prone to crystallization in 15% PEG8000 with a pH range of 6.5–8.5. Large cube crystals were obtained after optimization. However, they diffracted poorly, usually at 8–10 Å resolution. Moreover, a skin was formed in the crystallization drop, which made it difficult to harvest the crystals. The breakthrough was made with deglycosylated proteins, three different forms of crystals were obtained. The cylinder crystals diffracted poorly, with the best diffraction to 6.8 Å. The optimization could not decrease the nucleation of crystals, and a skin was also found on the crystallization drop. Rodlike crystals were so fragile that they disappeared when harvesting. Cuboid crystals obtained using seeding method appeared in 2 days and grew to full size in 2 weeks using the hanging-drop vapor-diffusion method. The well buffer contained 0.1 M Tris, pH 8.5 and 2.0 M ammonium phosphate.

### Diffraction data collection and structure determination

All data sets were collected on beamlines BL17U and BL19U at the Shanghai Synchrotron Radiation Facility (SSRF) or BL5A at the Photon Factory (KEK). Data were indexed, integrated and scaled using HKL2000^55^, and processed according to CC_1/2_^56^.

For no homologous protein of MRJP1 was found in the Protein Data Bank (PDB), we tried to solve the MRJP1 oligomer structure by multiple isomorphous replacement (MIR) or multiple-wavelength anomalous diffraction (MAD) method. More than 100 MRJP1 oligomer crystals, soaked in the reservoir solution mixed with different heavy metals or haloids such as Pt, Au, Hg, Pb, Br, or I under variable concentration and time, were screened. Some crystals after heavy metal soaking still displayed poor or even no diffraction, and the others had good diffraction but with weak anomalous signal of heavy metal. We also tried the co-crystallization of MRJP1 and different concentration of several kinds of heavy metals or haloids. Nevertheless these crystals with strong diffraction had no specific heavy metal ions or haloids bound. Moreover, we also expressed the selenomethionyl MRJP1 in both *E. coli* and yeasts, but we failed in the generation of diffraction-quality crystals.

After the failure of extensive trials to solve the phase by MIR or MAD methods, we searched for the weak homology of MRJP1 and found a structure of a Yellow family protein LJM11 from the saliva of the sand fly *lutzomyia longipalpiss*^38^. The sequence homology was low between LJM11 and MRJP1 with a value of 16.9%. Using the monomer structure of LJM11 (PDB: 3Q6K) as the search model, an initial molecular replacement (MR) solution of MRJP1 was found by BALBES^57^ after extensive trials of different resolution and space groups (*H*32 or *H*3). The best solution found two monomers with an MR score of 2.20 and an *R*_work_/*R*_free_ value of 0.538/0.568 for space group *H*32. After removing the inappropriate main and side chains in the model using the program COOT^58^, the optimized monomer structure was used to find the right position by program BALBES again. The resulting model was then fed back to the program IPCAS^59^ to combine single isomorphous replacement phasing and partial structure information to break the phase ambiguity intrinsic in the MR method. The primitive density map has a relatively clear outline, with many recognizable alpha-helix bundles.

Then we iteratively refined and built the model using CNS^60^, IPCAS^59^, the CCP4 suite^61^ and the autobuild program in the Phenix^62^. According to the constraints of non-crystallographic symmetry with twofold, the non-rational main and side chains were adjusted on the electron density map using the program COOT. After more than 100 cycles of manual rebuilding and refining, the best solution stopped at an *R*_work_/*R*_free_ value of 0.32/0.36. At this point, the structures of two MRJP1 molecules were well fitted into electron-density maps with clear α-helices and β-strand bundles. Interestingly, two anti-parallel α-helices and four blobs density were found and fit to two apisimin and four 24-methylenecholesterol molecules, respectively. After more than 40 rounds of position modulation and five rounds of simulated annealing correction, we continued to modulate the non-rational main and side chains to make R factor, Φ–Ψ angle and B factor lying in the allowed regions. Finally, the crystal structure of MRJP1 oligomers was improved by non-crystallographic symmetry restraints. The final structure was refined to a resolution of 2.65 Å in the space group *H*32, with an *R*_work_ of 23.9% and an *R*_free_ of 27.5% (Table 1). The whole-MRJP1-Apisimin-24-methylenecholesterol ternary structure was obtained by a twofold axis using a symmetric operation.

All structural figures in this article were drawn by using the PyMOL program^63^. The Adaptive Poisson-Boltzmann Solver^64^ combined with PDB2PQR was used for molecular electrostatics calculations.

### Analytical ultracentrifugation

Sedimentation velocity (SV) experiments were performed in a Beckman/Coulter XL-I analytical ultracentrifuge using double-sector or six-channel centerpieces and sapphirine windows. Before SV experiments, an additional protein purification step was applied using size exclusion chromatography in buffer B containing 20 mM Tris-HCl (pH 8.0) and 150 mM NaCl. The experiments were conducted at 42,000 rpm and 4 °C using interference detection and double-sector cells loaded at approximate 0.5 mg/mL for MRJP1 proteins. The SV data were analyzed using the SEDFIT and SEDPHAT programs^65^.

### Small angle X-ray scattering experiments

Small angle X-ray scattering (SAXS) data (Supplementary Table 2 and Supplementary Data 1) were collected at the BioSAXS station (1W2A) of the BSRF or beamline BL19U2 of the SSRF, using previously published methods^66^. Briefly, the proteins were subjected to size exclusion chromatography with a buffer containing 20 mM sodium phosphate (pH 8) and 150 mM NaCl. The protein concentrations were in a gradient concentration of 0.1, 0.2, 0.5, 1, 2, 5, 10 mg/mL, and the data of the protein samples were collected at 1.54 Å with a distance of 1.64 m from the detector. Data collection time of 5 min was used for all samples split into two equal time frames to assess and remove effects from radiation damage to the samples. Individual data were processed by FIT2D^67^. The average of the scattering from the buffer alone before and after each sample was used for background subtraction. The theoretical scattering curves were fitted to the experimental scattering curve using MES.

### Synchrotron radiation circular dichroism spectra

The Synchrotron radiation circular dichroism (CD) spectra of MRJP1 proteins were collected on beamline 4B8 of the Beijing Synchrotron Radiation Facility. All proteins were changed to a buffer solution 20 mM sodium phosphate (pH 8.0), filtered and accurately weighed. All spectra were recorded three times using a 0.007 mm optical path length quarts SUPRASIL cell, over a wavelength range of 175–255 nm in 1 nm steps and a dwell time of 1 s per wavelength point. A pure solvent baseline was measured with the same cell and subtracted. CD spectra were also measured at 2 °C or 5 °C intervals from 5 °C to 85 °C. The sample was equilibrated 5 min at each temperature prior to the measurement.

All spectra were processed by the CDtool software package. The machine unit (mdeg) was converted into the per residue molar absorption unit, delta epsilon (Δε) in M cm^-1^, by normalization with respect to protein concentration and path length. Secondary structure content analysis of the spectra was based on the CDSSTR reference set 6 from the software package CDPro^68^.

### Isothermal titration calorimetry

Experiments were performed on a Nano ITC instrument (TA Instruments) at 20 °C. An additional protein purification step was applied using size exclusion chromatography in buffer B before applying to ITC. MRJP1 oligomer was diluted to 2 mg/mL using buffer B. A total of 200 mM serotonin was dissolved in buffer B and adjusted to pH 8.0 with NaOH. A total of 2 mM serotonin was obtained by diluting 200 mM stock solution with buffer B. A total of 2 mM serotonin in the syringe was titrated against 2 mg/mL MRJP1 proteins in the cell. ITC data were processed with NanoAnalyze software (TA Instruments).

### Isolation of small molecules from MRJP1 oligomer

A total of 5 mL 2 mol/L NaOH was added to 1 g purified MRJP1 oligomer, then the solution was extracted with 30 mL n-hexane for three times. The combined n-hexane part was washed by water, then was evaporated to remove the organic solvents.

### GC–MS of 24-methylenecholesterol

The small molecule isolated from the purified MRJP1 oligomer was confirmed by EI-GC–MS analysis via a QP-2010 mass spectrometer (Tokyo, Japan). The GC was equipped with a DB-5MS (25 m × 0.25 mm × 0.25 µm) capillary column (Agilent, Santa Clara, CA, USA). The temperature of the injector was kept at 300 °C, and 1.0 µL of the sample solution was injected in high pressure and splitless mode. The flow control mode was a constant linear velocity (36.8 cm/s). Helium (purity ≥99.999%) at 1.0 mL/min was used as the carrier gas. The oven temperature was programmed as follows: the initial temperature was 100 °C (maintained for 1 min), 10 °C/min ramp, 150 °C, 30 °C/min, 290 °C for 15 min. The running time for each assay was ~26 min.

The conditions were set as follows: mass spectra and retention times were obtained in EI mode (70 eV), with an ion source temperature of 200 °C, and a transfer line temperature of 280 °C. MS data were acquired in the scan mode (*m/z* 40–1000) for quantization with 4 min of solvent delay. The GC–MS Solution Version 2.40 and NIST database were used for data processing and compound searching, respectively. The data are consistent with the standard spectra in the website (http://www.massbank.jp/jsp/FwdRecord.jsp?type=disp&id=JP008184) with molecular formula C_28_H_46_O (MS *m/z*: 398).

### NMR spectroscopic data for 24-methylenecholesterol

The small molecule isolated from the purified MRJP1 oligomer was further confirmed by NMR analysis. NMR spectra in CDCl_3_ were recorded on a Bruker AV III HD-600 instrument for ^1^H and ^13^C using standard pulse programs and acquisition parameters. Chemical shifts were reported in δ (ppm) referencing to the NMR solvent used. The data were consistent with the references^37^ and^69^.

^1^H NMR (600 MHz, CDCl_3_) *δ*: 5.36 (d, *J* = 4.8 Hz, 1H, 6-H), 4.71 (s, 1H, 28-H), 4.66 (d, *J* = 1.2 Hz, lH, 28-H), 3.55–3.50 (m, lH, 3α-H), 2.31–2.28 (m, 1H), 2.25–2.21 (m, 2H), 2.12–2.07 (m, 1H), 2.03–1.85 (m,2H), 1.61–1.52 (m, 4H), 1.51–1.49 (m, 2H), 1.45–1.40 (m, 3H), 1.31–1.25 (m, 2H), 1.25 (s, 2H), 1.19–1.12 (m, 3H), 1.11–1.05 (m, 2H), 1.03 (d, *J* = 6.6 Hz, 3H, 26-H_3_), 1.02 (d, *J* = 7.2 Hz, 3H, 27-H_3_), 1.01 (s, 3H, 19-H_3_), 0.99–0.97 (m, 1H), 0.95 (d, *J* = 6.6 Hz, 3H, 21-H_3_), 0.93–0.87 (m, 1H), 0.68 (s, 3H, 18-H_3_).

^13^C NMR (150 MHz, CDCl_3_) *δ*: 156.9 (24-C), 140.8 (5-C), 121.7 (6-C), 106.0 (28-C), 71.8 (3-C), 56.8 (14-C), 56.1 (17-C), 50.2 (9-C), 42.4 (4-C), 42.4 (13-C), 39.8 (12-C), 37.3 (1-C), 36.5 (10-C), 35.8 (20-C), 34.8 (23-C), 33, 9 (25-C), 32.0 (7-C), 31.9 (8-C), 31.7 (2-C), 31.0 (22-C), 28.2 (16-C), 24.3 (15-C), 22.0 (27-C), 21.9 (26-C), 21.1 (11-C), 19.4 (19-C), 18.7 (21-C), 11.9 (18-C).

### Data availability

The accession number for the coordinates of the MRJP1-apisimin-24-methylenecholesterol complex reported in this paper is PDB: 5YYL. 24-methylenecholesterol has been added to the PDB component dictionary with code 94R. Further data are available from the corresponding author upon reasonable request.

## Electronic supplementary material

Supplementary Information

Description of Additional Supplementary Files

Supplementary Data 1

Supplementary Movie 1
