# Supplementary material for: Architecture of the native major royal jelly protein 1 oligomer
Source: Nat Commun. 2018 Aug 22;9:3373. doi: 10.1038/s41467-018-05619-1 (PMC6105727; doi:10.1038/s41467-018-05619-1)
Supplement: Supplementary file 1 — Supplementary Information [file 41467_2018_5619_MOESM1_ESM.pdf]

Architecture of the Native Major Royal Jelly Protein 1 Oligomer

W. Tian, M. Li, H. Guo, W. Peng et al.

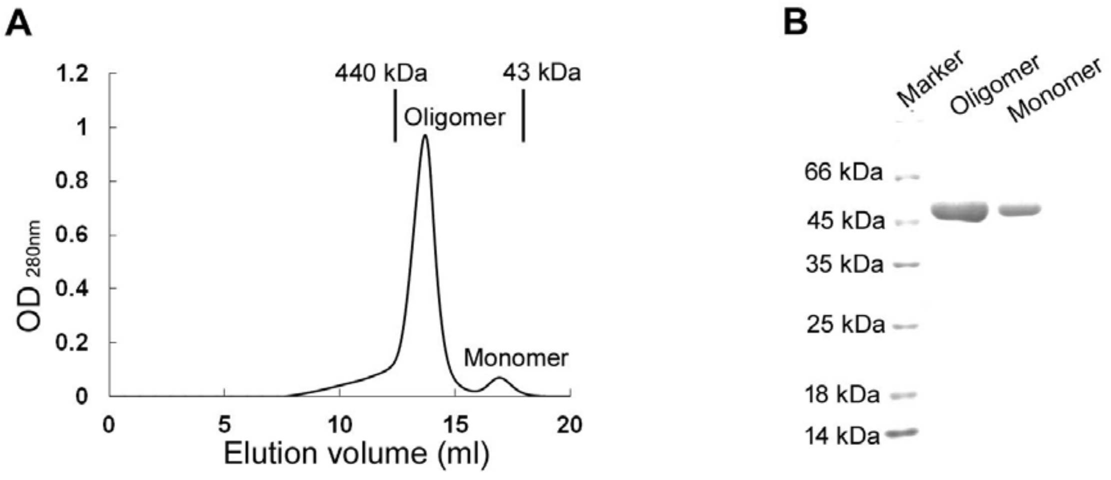

C

Match to	Sequence name	Nominal mass (Mr)	Calculated *pI* value	Score	Sequence Coverage
gi|58585098	Major royal jelly protein 1 [*Apis mellifera*]	49311	5.10	12755	84%

D

```
  1 MTRLFMLVCL GIVCQGTTGN ILRGESLNKS LPILHEWKFF DYDFGSDERR
 51 QDAILSGEYD YKNNYPSDID QWHDKIFVTM LRYNGVPSSL NVISKKVGDG
101 GPLLQPYPDW SFAKYDDCSG IVSASKLAID KCDRLWVLDS GLVNNTQPMC
151 SPKLLTFDLT TSQLLKQVEI PHDVAVNATT GKGRLSSLAV QSLDCNTNSD
201 TMVYIADEKG EGLIVYHNSD DSFHRLTSNT FDYDPKFTKM TIDGESYTAQ
251 DGISGMALSP MTNNLYYSPV ASTSLYYVNT EQFRTSDYQQ NDIHYEGVQN
301 ILDTQSSAKV VSKSGVLFFG LVGDSALGCW NEHRTLERHN IRTVAQSDET
351 LQMIASMKIK EALPHVPIFD RYINREYILV LSNKMQKMVN NDFNFDDVNF
401 RIMNANVNEL ILNTRCENPD NDRTPFKISI HL
```

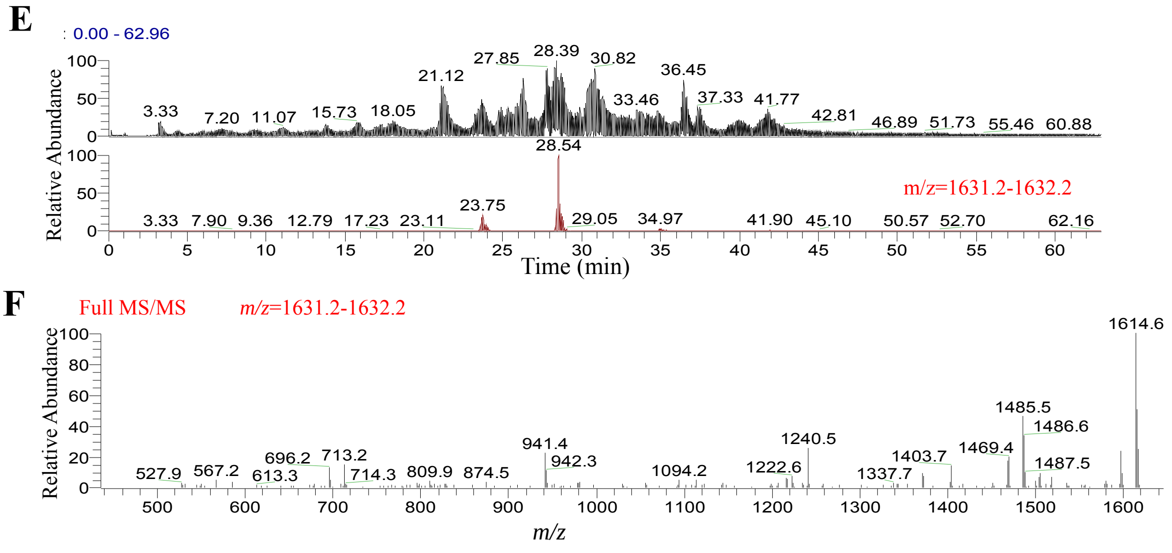

Supplementary Figure 1. The separation and mass spectrometric (MS/MS) identification of the purified native MRJP1.

Size exclusion chromatography (**A**) and SDS-PAGE (**B**) of MRJP1 oligomer and monomer on a Superdex 200 10/300 GL column (GE Healthcare). In sieve column, peak positions for two standard proteins were indicated as black lines on the top. (**C**) Table of the main characteristics of the digested peptides from MRJP1 oligomer analyzed by MALDI-TOF identified in database. (**D**) MRJP1 sequence with access number 58585098 (new number: NP_001011579.1) indicated the 84% coverage of the peptides (underline/**bold**). (**E**) Total ion chromatograms and extracted ion (*m/z*=1631.2-1632.2) chromatogram of digested MRJP1 oligomer with LC-MS/MS. (**F**) One example of MS/MS spectra of the peptide (*m/z*=1631.2-1632.2). Note that the glycosylated MRJP1 oligomer was used here.

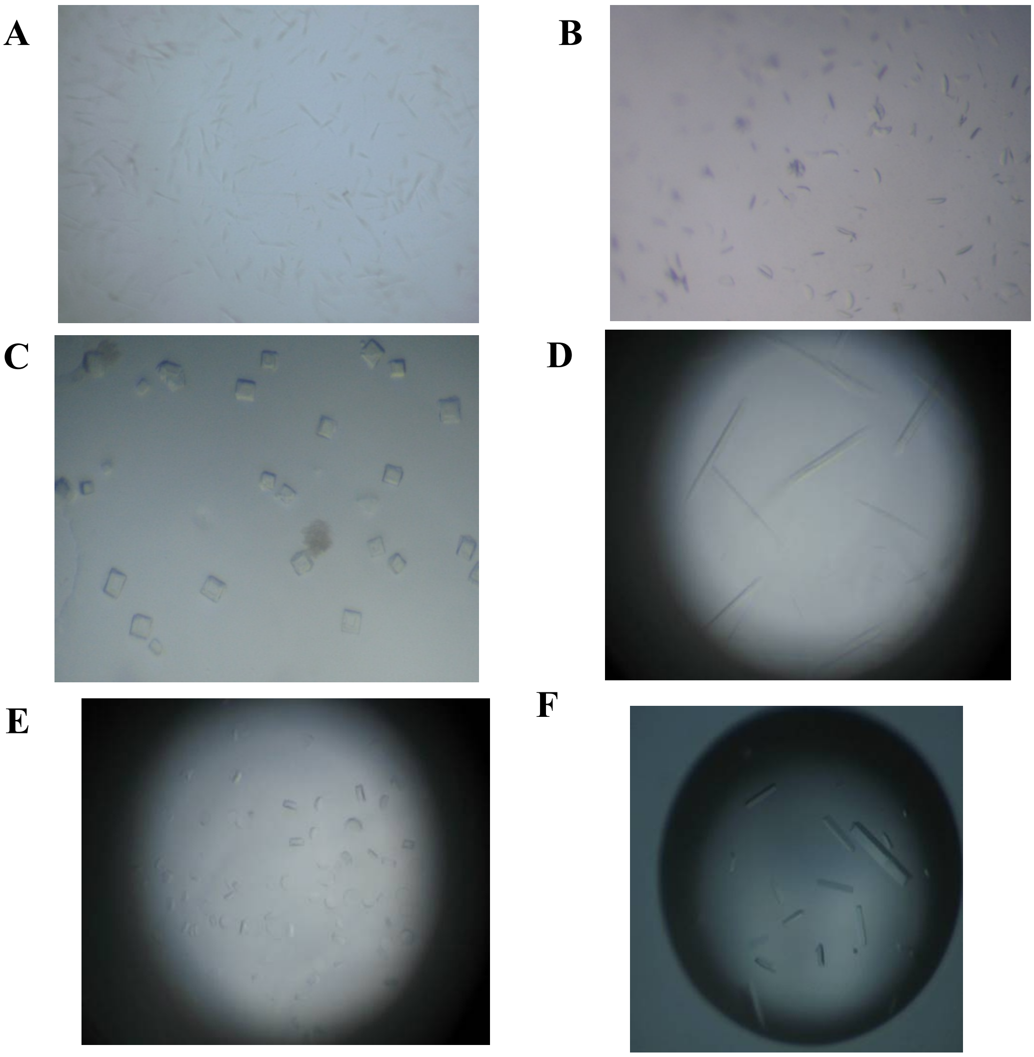

Supplementary Figure 2. **The crystals of native MRJP1 oligomers.**

(**A**) Needle-like crystals obtained from initial screening. (**B**) Crescent crystals after optimal crystallization conditions. (**C**) Cubic crystals of MRJP1 oligomer with lysine methylation according to the protocol [1]. Crystals of MRJP1 oligomers after deglycosylation (**D-F**). (**D**) Rodlike crystals. (**E**) Cylindrical crystals. (**F**) Cuboid crystals after seeding. Cuboid crystals were used to collect diffraction data.

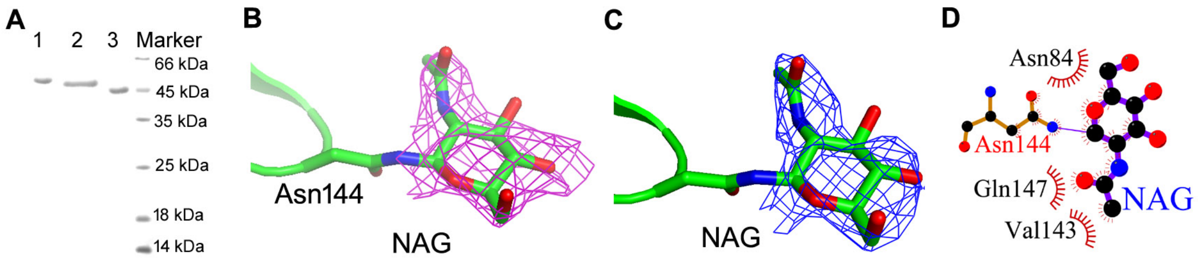

Supplementary Figure 3. **Native MRJP1 oligomer is partly deglycosylated by PNGase F under mild condition.**

(**A**) Electrophoretic pattern of natural and denatured MRJP1 treated with peptide:N-glycanase (PNGase) F. Lane 1. Natural MRJP1 oligomer before digestion. Lane 2. Natural MRJP1 oligomer digested by PNGase F at 4ºC. Lane 3. Denatured MRJP1 treated with PNGase F at 37ºC. (**B**) 2mF_o−DFc electron density map of N-acetylglucosamine (NAG) at 1.0 σ. (**C**) Composite simulated-annealing mF_o−DFc "omit" electron density map of NAG at 2.0 σ. (**D**) The NAG is protected by the surrounding residues, preventing from cleavage by PNGase F.

A

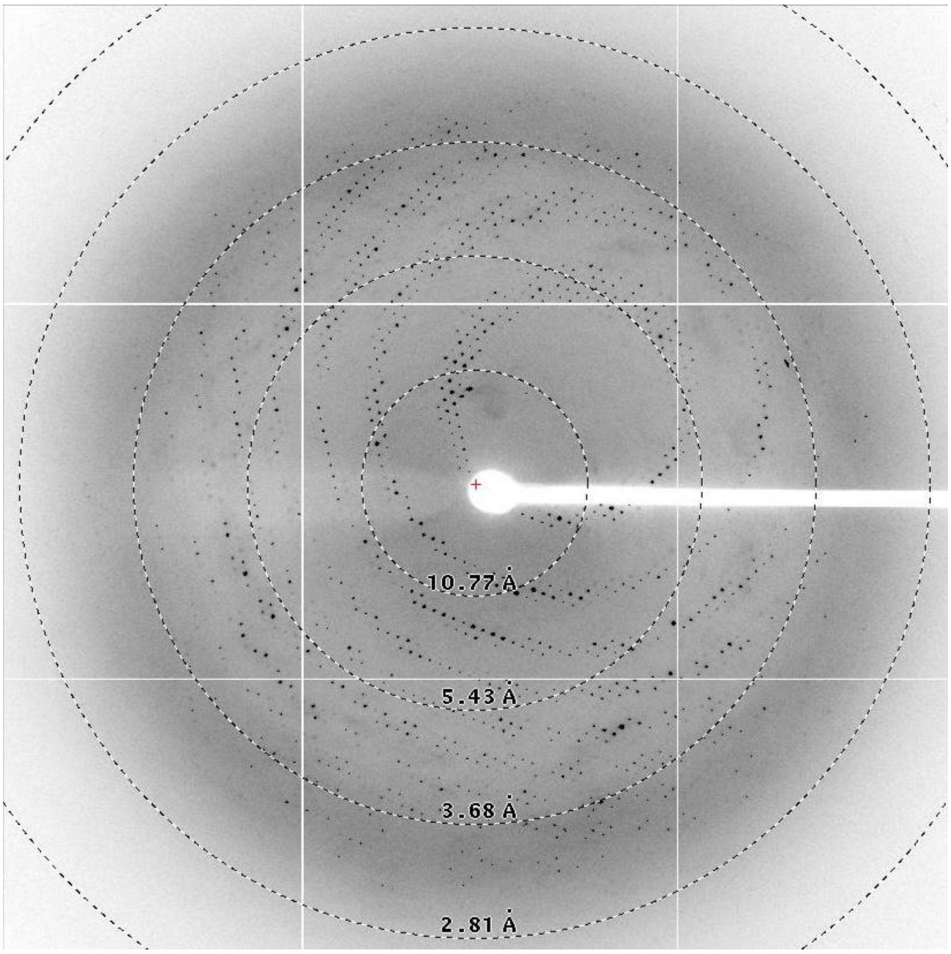

B

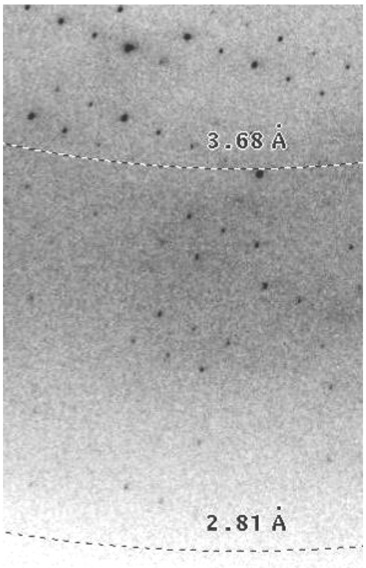

C

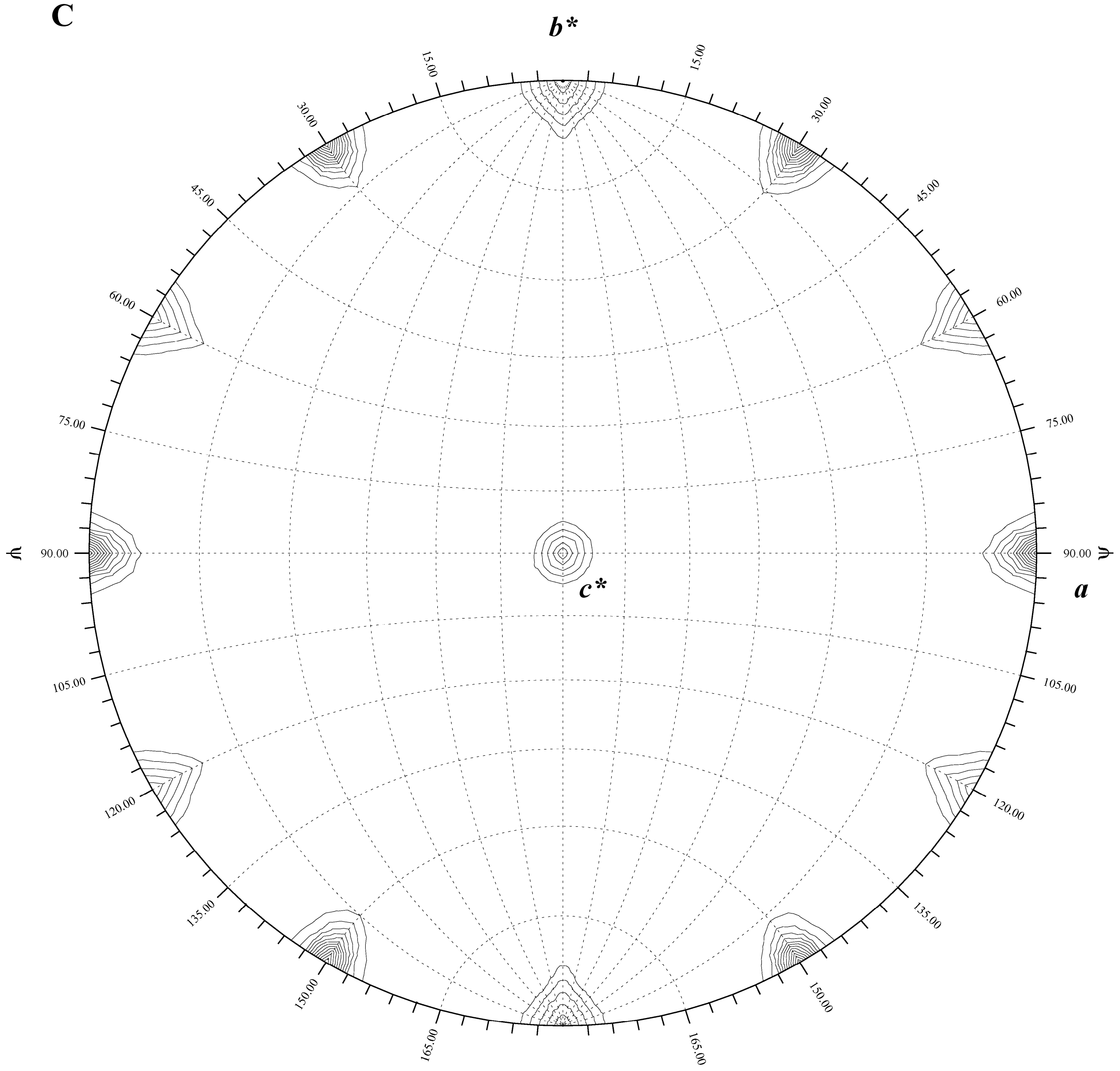

κ = 180.00

Search Information :
Search Angle : Polar, Convention : XYK
Search Limits :

```
  Angle 1 :     0.00 180.00   3.00 61
  Angle 2 :     0.00 180.00   3.00 61
  Angle 3 :   180.00 180.00   0.00  1
Avg., Sd  :   231.86   35.50
Min., Max :   156.40 1000.02
```

Radius : 25.0A, IGEVAL : 2
Resolution : 10.00 - 3.50A
Orthogonalization : AXABZ
Origin is removed
A OBS File : lm1-4.sca

A Cell : 211.57 211.57 149.97 90.00 90.00 120.00
A Cutoff : 1.00 1.00 0.00
No. of Refl. : 15320
Self-Rotation Search
Cutoff : 1.50
No. of large terms : 3267
Rcutoff : 1.0 20.0
Contour Levels:

```
  Contour # 1 :   400.0 1000.0   50.0  1
```

Supplementary Figure 4. X-ray diffraction image of MRJP1 oligomer crystals at 2.65 Å and its self-rotation function.

Representative X-ray diffraction image (**A**) and its enlarged picture of the high

resolution area (**B**). Stereographic projection (**C**) showing the ordinary self-rotation function of MRJP1 oligomer. Reflection data between 10 and 3.5 Å resolution were used in the calculation with the program GLRF [2]. The radius of integration was 25 Å. The result shows that it exists a non-crystallographic symmetry two-fold.

A

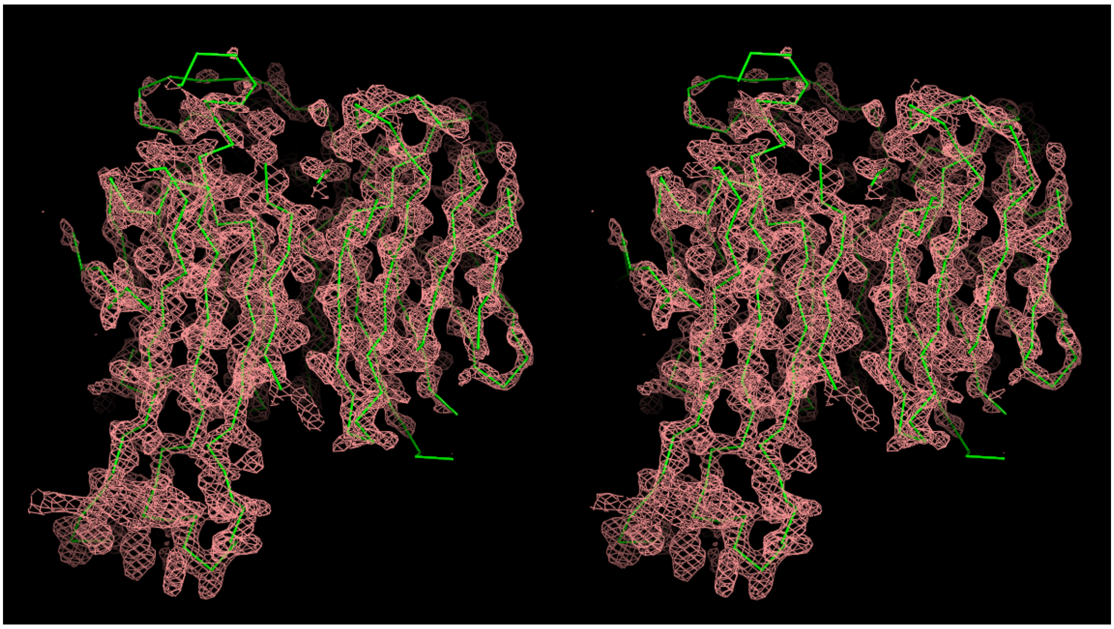

B

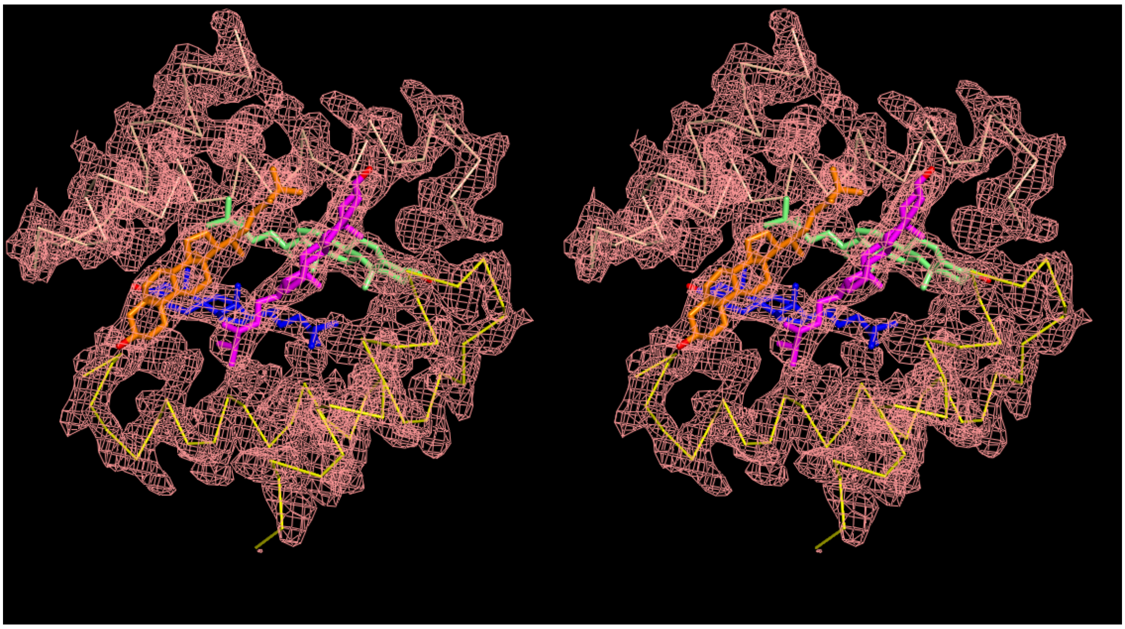

Supplementary Figure 5. Representative electron density maps of MRJP1-apisimin- 24-methylenecholesterol (Osl) complex in one asymmetric unit.

(**A**) Representative experimental electron density map of one MRJP1 molecule. The electron density (2*mFo-DFc*) is contoured at 2.0 σ. (**B**) Experimental electron density map (2*mFo-DFc*) of two apisimin (yellow or wheat) and four Osl (blue, purple, orange, or lime sticks) molecules, contoured at 1.5 σ.

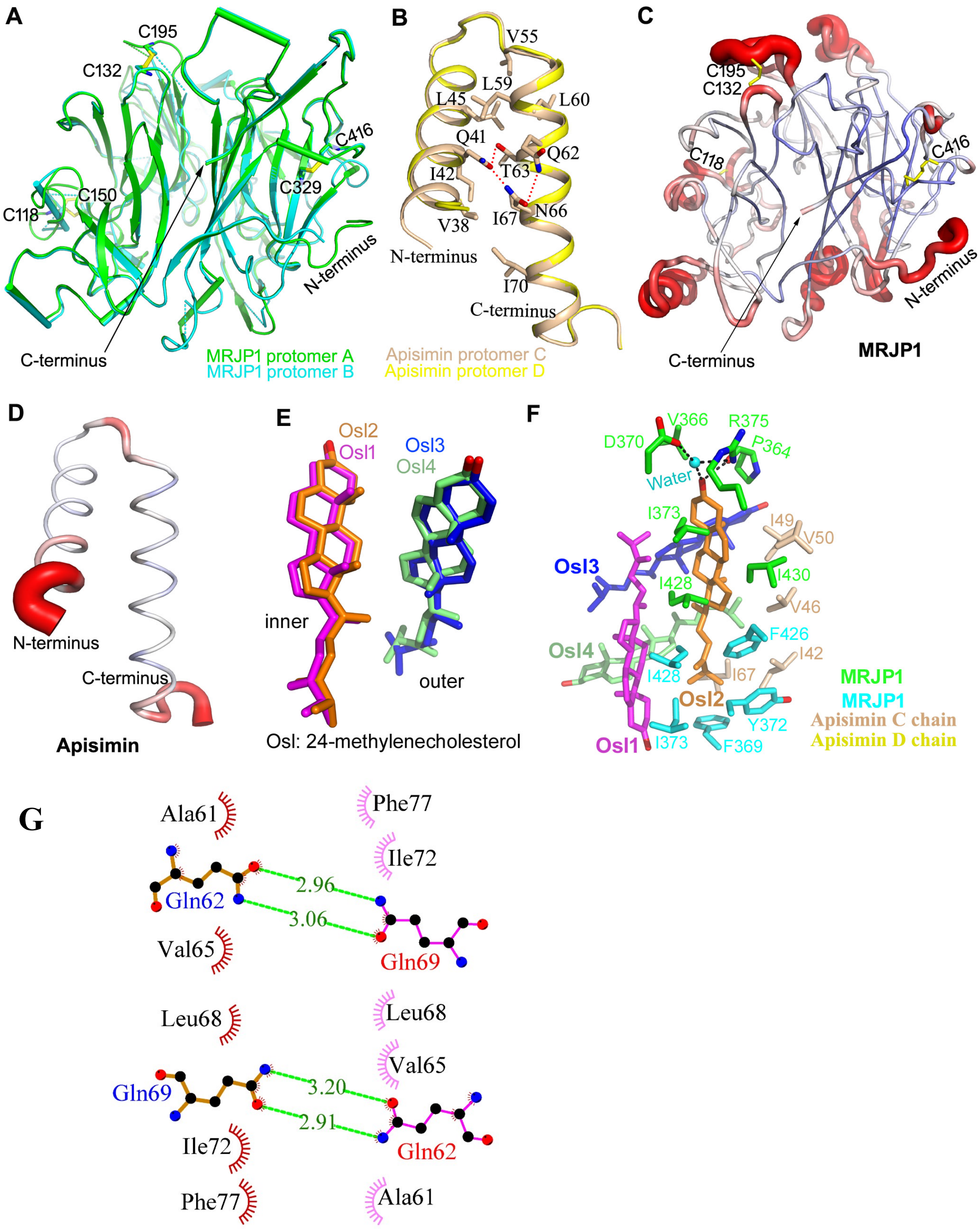

Supplementary Figure 6. **Structural differences of MRJP1, apisimin and Osl in the asymmetric unit.**

Structures alignment of the two MRJP1 (**A**) and apisimin (**B**) protomers in the asymmetric unit, respectively. Disulfide bonds are shown in yellow and dashed lines indicate residues that have not been resolved in the structures of figure **A**. Overall B-factors of MRJP1 (**C**) and apisimin (**D**), color-coded on the basis of the calculated B-factors. The colors range from blue to red corresponding to increasing fluctuations. The alignment of four Osl by the non-crystallographic symmetry two-fold (**E**) and interactions of Osl2 (**F**). (**G**) The interactions between apisimin and the neighbouring symmetry-generated apisimin molecule using Ligplot [3]. Hemispheres represent

hydrophobic interactions and lines represent polar interactions. Residues from two protomers are shown on the left and right, respectively. All residues involved in the hydrophobic interactions are shown in black. For hydrophilic interactions, residues are shown in red or blue.

A

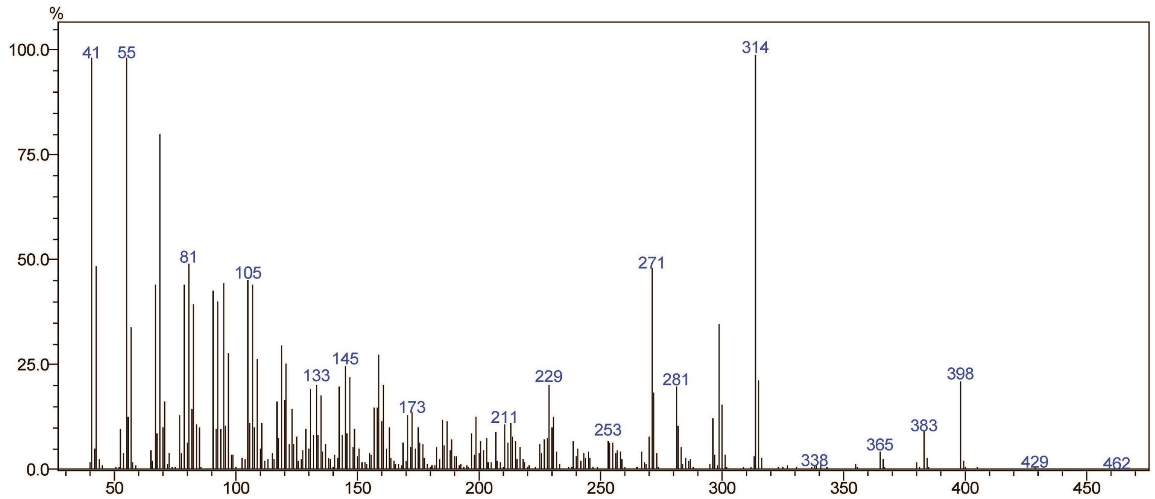

%
100.0
75.0
50.0
25.0
0.0
41
55
81
105
133
145
173
211
229
253
271
281
314
338
365
383
398
429
462
50
100
150
200
250
300
350
400
450

B

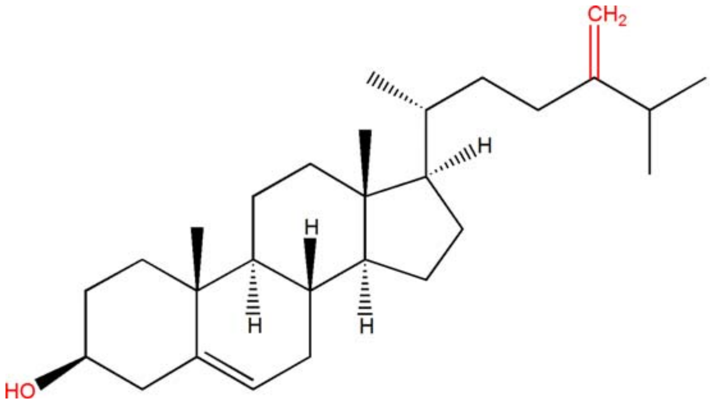

CH2
H
H
H
H
HO

C

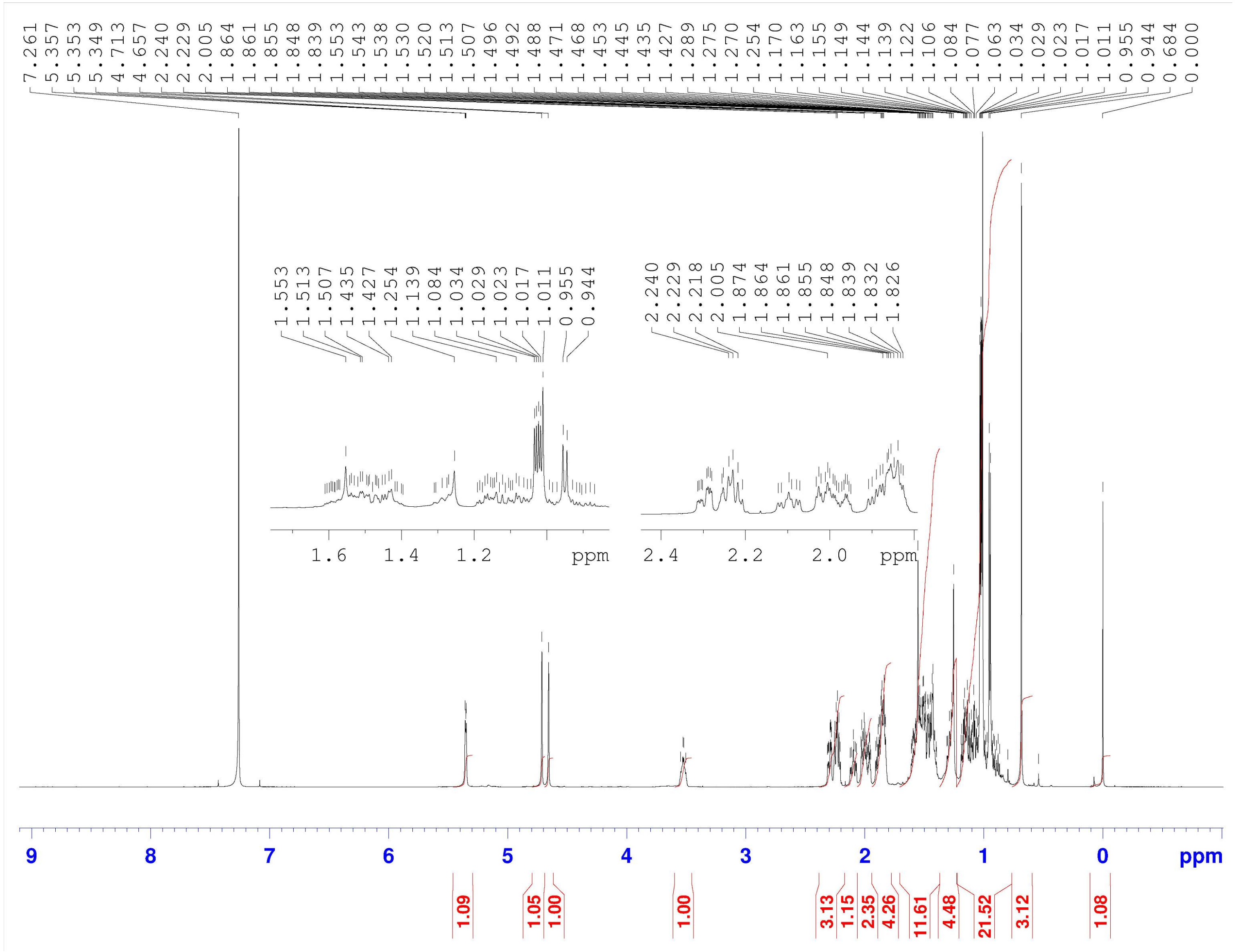
7.261 5.357 5.353 5.349 4.713 4.657 2.240 2.229 2.005 1.864 1.861 1.855 1.848 1.839 1.553 1.543 1.538 1.530 1.520 1.513 1.507 1.496 1.492 1.488 1.471 1.468 1.453 1.445 1.435 1.427 1.289 1.275 1.270 1.254 1.170 1.163 1.155 1.149 1.144 1.139 1.122 1.106 1.084 1.077 1.063 1.034 1.029 1.023 1.017 1.011 0.955 0.944 0.684 0.000
1.553 1.513 1.507 1.435 1.427 1.254 1.139 1.084 1.034 1.029 1.023 1.017 1.011 0.955 0.944
1.6 1.4 1.2 ppm
2.240 2.229 2.218 2.005 1.874 1.864 1.861 1.855 1.848 1.839 1.832 1.826
2.4 2.2 2.0 ppm
9 8 7 6 5 4 3 2 1 0 ppm
1.09 1.05 1.00 1.00 3.13 1.15 2.35 4.26 11.61 4.48 21.52 3.12 1.08

D

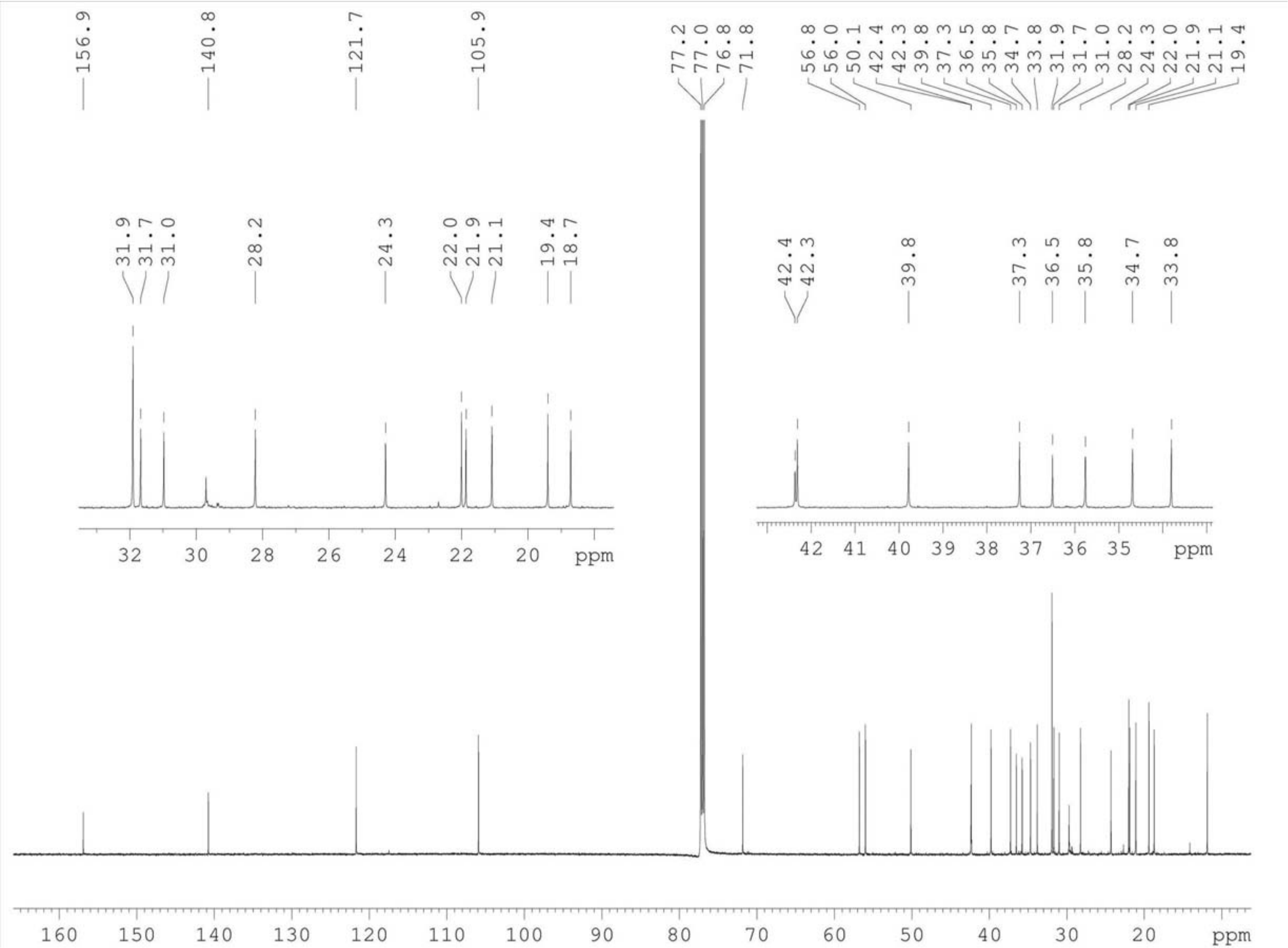

156.9
140.8
121.7
105.9
77.2
77.0
76.8
71.8
56.8
56.0
50.1
42.4
42.3
39.8
37.3
36.5
35.8
34.7
33.8
31.9
31.7
31.0
28.2
24.3
22.0
21.9
21.1
19.4
18.7
ppm

Supplementary Figure 7. **Identification of Osl purified from the MRJP1 oligomer.**

EI-GC/MS mass spectra (**A**) and structure (**B**) of Osl (molecular formula $C_{28}H_{46}O$, MS *m/z*: 398). The mass spectra are consistent with the standard spectra in the website (http://www.massbank.jp/jsp/FwdRecord.jsp?type=disp&id=JP008184). **C**. ^{1}H NMR spectra of Osl purified from the MRJP1 oligomer at 600 MHz. **D**. ^{13}C NMR spectra of Osl purified from the MRJP1 oligomer at 150 MHz. The proton and carbon magnetic resonance spectra are consistent with those in the reference [4].

A

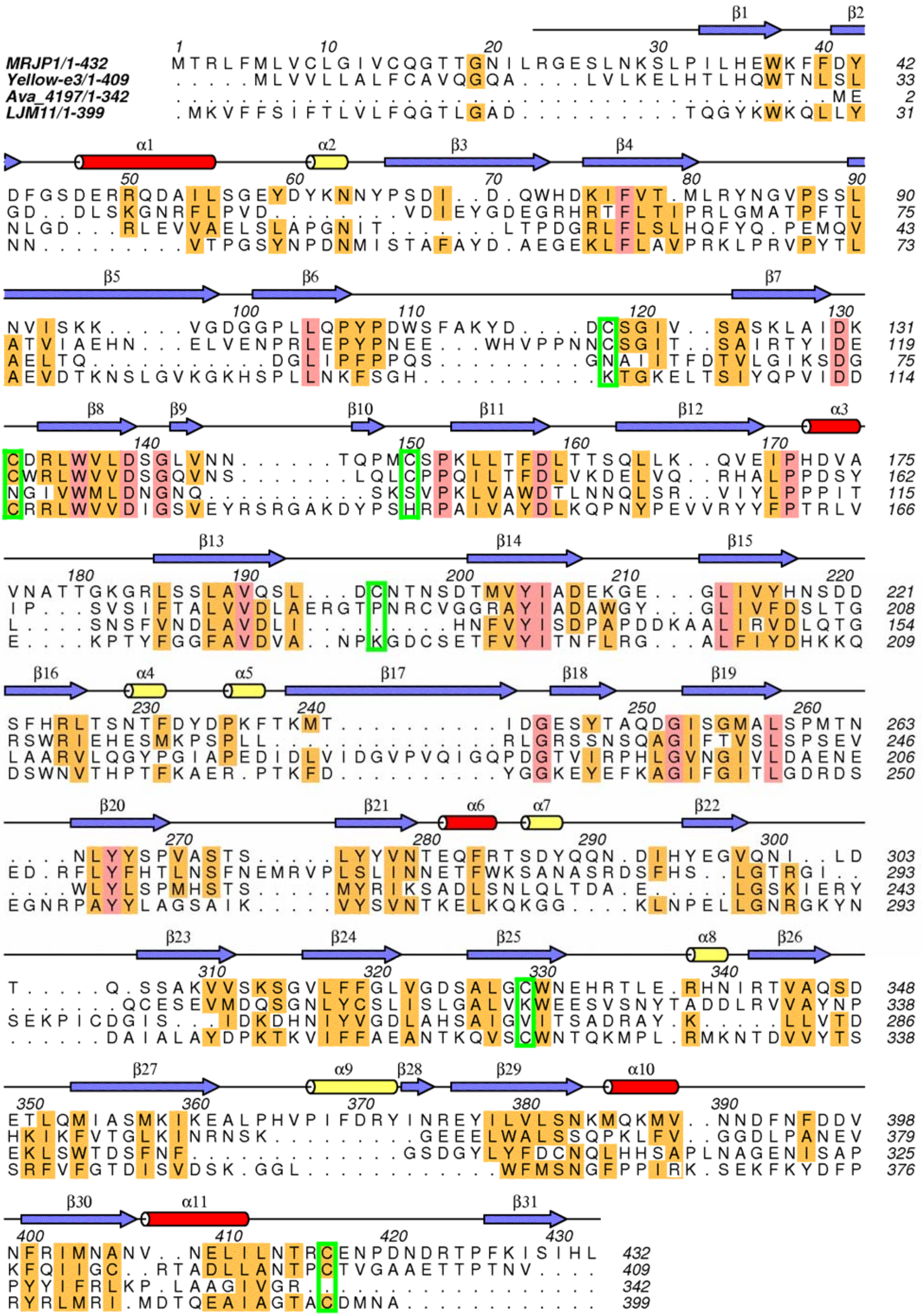

β1 β2
MRJP1/1-432 MTRLFMLVCLGIVCQGTTGNILRGESLNKSLPILHEWKFFDY 42
Yellow-e3/1-409 .....MLVVLLALFCAVQGQA....LVLKELHTLHQWTNLSL 33
Ava_4197/1-342 ....ME 2
LJM11/1-399 .MKVFFSIFTLVLFQGTLGAD..........TQGYKWKQLLY 31
α1 α2 β3 β4
DFGSDERRQDAILSGEYDYKNNYPSDI..D.QWHDKIFVT.MLRYNGVPSSL 90
GD..DLSKGNRFLPVD.......VDIEYGDEGRHRTFLTIPRLGMATPFTL 75
NLGD...RLEVVAELSLAPGNIT.......LTPDGRLFLSLHQFYQ.PEMQV 43
NN.........VTPGSYNPDNMISTAFAYD.AEGEKLFLAVPRKLPRVPYTL 73
β5 β6 β7
NVISKK.....VGDGGPLLQPYPDWSFAKYD....DCSGIV..SASKLAIDK 131
ATVIAEHN...ELVENPRLEPYPNEE...WHVPPNNCSGIT..SAIRTYIDE 119
AELTQ...........DGLIPFPPQS.........GNAIITFDTVLGIKSDG 75
AEVDTKNSLGVKGKHSPLLNKFSGH...........KTGKELTSIYQPVIDD 114
β8 β9 β10 β11 β12 α3
CDRLWVLDSGLVNN......TQPMCSPKLLTFDLTTSQLLK..QVEIPHDVA 175
CWRLWVVDSGQVNS......LQLCPPQILTFDLVKDELVQ..RHALPPDSY 162
NGIVWMLDNGNQ.........SKSVPKLVAWDTLNNQLSR..VIYLPPPIT 115
CRRLWVVDIGSVEYRSRGAKDYPSHRPAIVAYDLKQPNYPEVVRYYFPTRLV 166
β13 β14 β15
VNATTGKGRLSSLAVQSL...DCNTNSDTMVYIADEKGE...GLIVYHNSDD 221
IP...SVSIFTALVVDLAERGTPNRCVGGRAYIADAWGY...GLIVFDSLTG 208
L....SNSFVNDLAVDLI.........HNFVYISDPAPDDKAALIRVDLQTG 154
E....KPTYFGGFAVDVA..NPKGDCSETFVYITNFLRG...ALFIYDHKKQ 209
β16 α4 α5 β17 β18 β19
SFHRLTSNTFDYDPKFTKMT..........IDGESYTAQDGISGMALSPMTN 263
RSWRIEHESMKPSPLL..............RLGRSSNSQAGIFTVSLSPSEV 246
LAARVLQGYPGIAPEDIDLVIDGVPVQIGQPDGTVIRPHLGVNGIVLDAENE 206
DSWNVTHPTFKAER.PTKFD..........YGGKEYEFKAGIFGITLGDRDS 250
β20 β21 α6 α7 β22
....NLYYSPVASTS.....LYYVNTEQFRTSDYQQN.DIHYEGVQNI..LD 303
ED.RFLYFHTLNSFNEMRVPLSLINNETFWKSANASRDSFHS..LGTRGI.. 293
....WLYLSPMHSTS.....MYRIKSADLSNLQLTDA.E.....LGSKIERY 243
EGNRPAYYLAGSAIK.....VYSVNTKELKQKGG....KLNPELLGNRGKYN 293
β23 β24 β25 α8 β26
T.....Q.SSAKVVSKSGVLFFGLVGDSALGCWNEHRTLE.RHNIRTVAQSD 348
.......QCESEVMDQSGNLYCSLISLGALVKWEESVSNYTADDLRVVAYNP 338
SEKPICDGIS...IDKDHNIYVGDLAHSAIGVITSADRAY.K.....LLVTD 286
......DAIALAYDPKTKVIFFAEANTKQVSCWNTQKMPL.RMKNTDVVYTS 338
β27 α9 β28 β29 α10
ETLQMIASMKIKEALPHVPIFDRYINREYILVLSNKMQKMV..NNDFNFDDV 398
HKIKFVTGLKINRNSK.........GEEELWALSSQPKLFV..GGDLPANEV 379
EKLSWTDSFNF.............GSDGYLYFDCNQLHHSAPLNAGENISAP 325
SRFVFGTDISVDSK.GGL............WFMSNGFPPIRK.SEKFKYDFP 376
β30 α11 β31
NFRIMNANV..NELILNTRCENPDNDRTPFKISIHL 432
KFQIIGC..RTADLLANTPCTVGAAETTPTNV.... 409
PYYIFRLKP.LAAGIVGR.................. 342
RYRLMRI.MDTQEAIAGTACDMNA............ 399

B

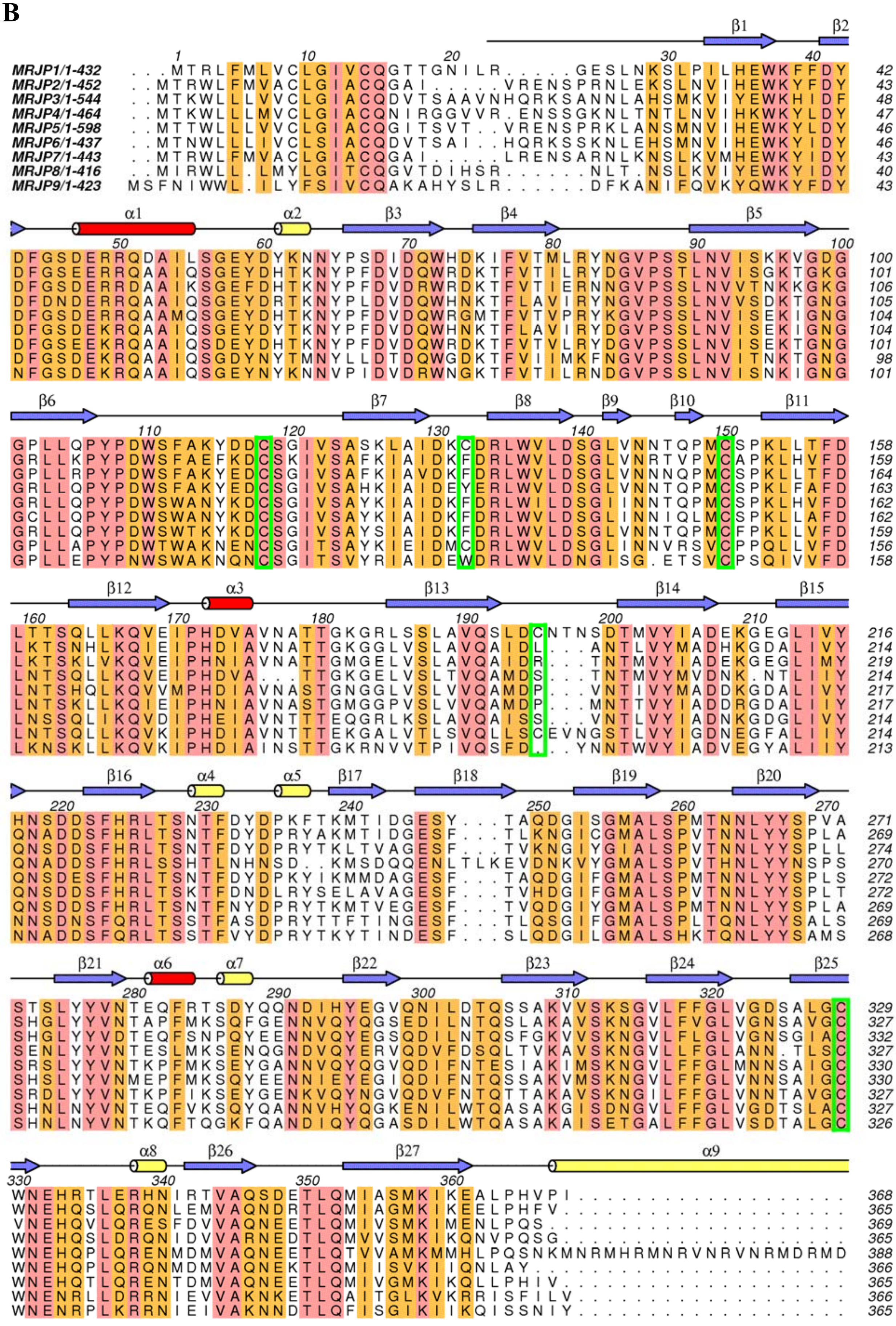
MRJP1/1-432
MRJP2/1-452
MRJP3/1-544
MRJP4/1-464
MRJP5/1-598
MRJP6/1-437
MRJP7/1-443
MRJP8/1-416
MRJP9/1-423
β1 β2 α1 α2 β3 β4 β5 β6 β7 β8 β9 β10 β11 β12 α3 β13 β14 β15 β16 α4 α5 β17 β18 β19 β20 β21 α6 α7 β22 β23 β24 β25 α8 β26 β27 α9

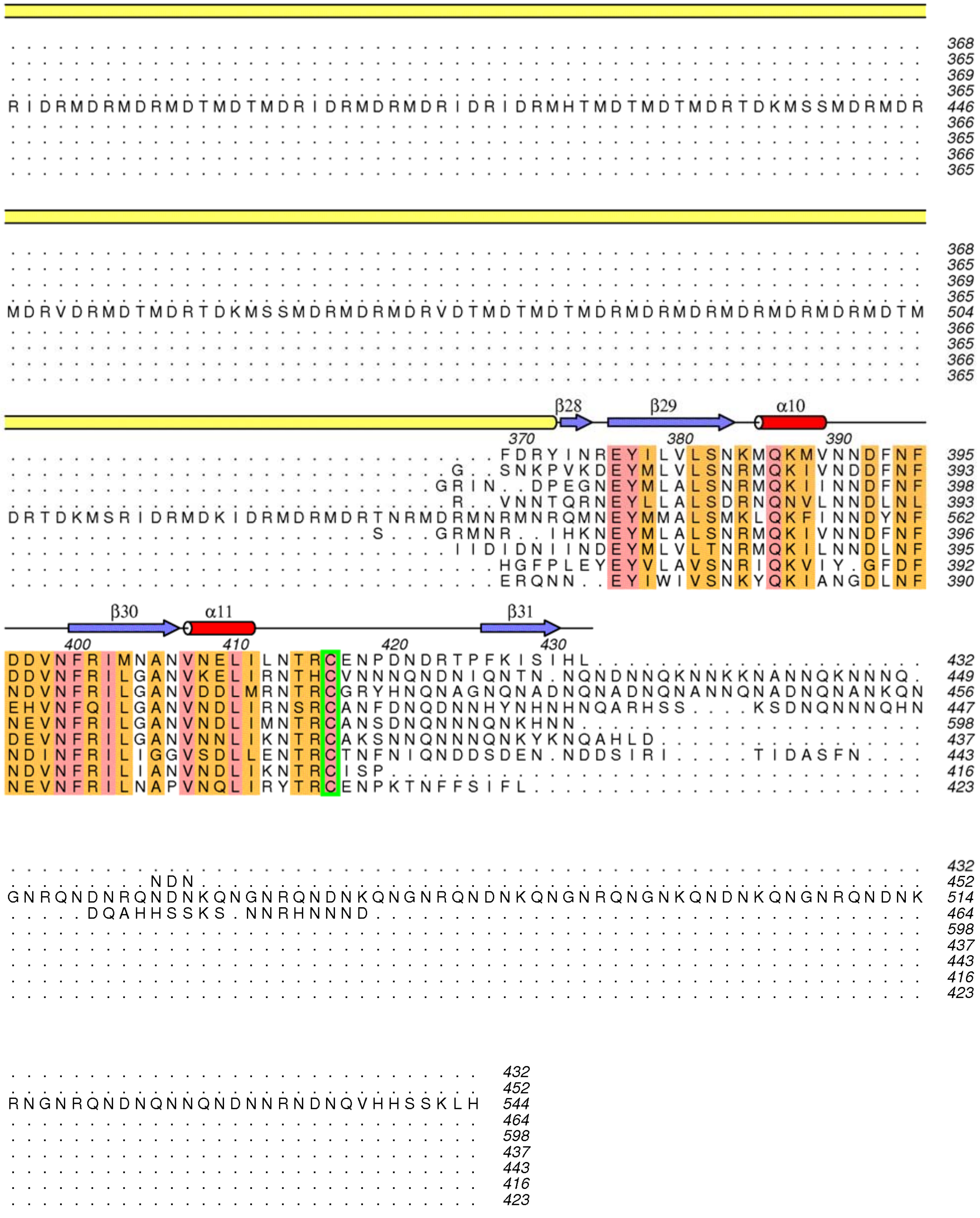
β28
β29
α10
β30
α11
β31

C

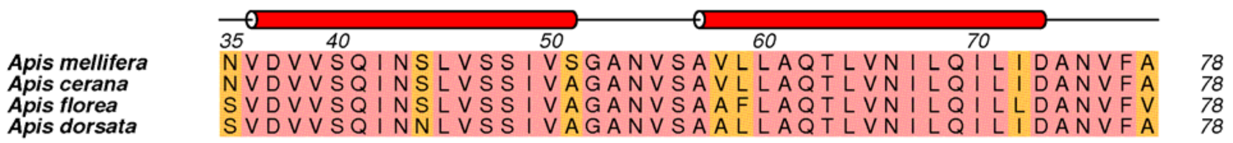
Apis mellifera
Apis cerana
Apis florea
Apis dorsata
NVDVVSQINSLVSSIVSGANVSAVLLAQTLVNILQILIDANVFA 78
NVDVVSQINSLVSSIVAGANVSAVLLAQTLVNILQILIDANVFA 78
SVDVVSQINSLVSSIVAGANVSAAFLAQTLVNILQILLDANVFV 78
SVDVVSQINNLVSSIVAGANVSAALLAQTLVNILQILIDANVFA 78

Supplementary Figure 8. Sequence alignments of Yellow family and apisimin homologues.

Sequence alignments of representative Yellow protein family (**A**), MRJP family (**B**) and apisimin homologues (**C**). Secondary structures are calculated by DSSP and indicated above the sequence alignment according to the structure of MRJP1 oligomer. The above numbers indicate residues for MRJP1 or apisimin. , and represent α-helices, 3_{10} helices and β-sheets, respectively. Green boxes indicate cysteine residues involved in the formation of disulfide bonds (also see Fig. 2). Residues with similarities or identities were shown in orange or magenta background, respectively. The sequence alignment was generated by ClustalW [5]. This figure was made by the program ALINE [6]. The sequences used are: MRJP1 from *Apis mellifera* (GenBank accession # - NP_001011579.1); Yellow-e3 from *Drosophila melanogaster* (NP_650288.1); Ava_4197 from *Anabaena variabilis* (strain ATCC 29413 / PCC 7937) (ABA23796.1); LJM11, 43.2 kDa salivary protein from *Lutzomyia longipalpis* (AAS05318.1). MRJP2, major royal jelly protein 2 precursor (NP_001011580.1); MRJP3, major royal jelly protein 3 precursor (NP_001011601.1); MRJP4, major royal jelly protein 4 precursor (NP_001011610.1); MRJP5, major royal jelly protein 5 precursor (NP_001011599.1); MRJP6, major royal jelly protein 6 precursor (NP_001011622.1); MRJP7, major royal jelly protein 7 precursor (NP_001014429.1); MRJP8, major royal jelly protein 8 precursor (NP_001011564.1); MRJP9, major royal jelly protein 9 precursor (NP_001019868.1).

For the sequences of **apisimin**, *Apis mellifera* (NP_001011582.1), *Apis cerana* (XP_016920890.1), *Apis florea* (XP_003692391.1), *Apis dorsata* (XP_006617609.1).

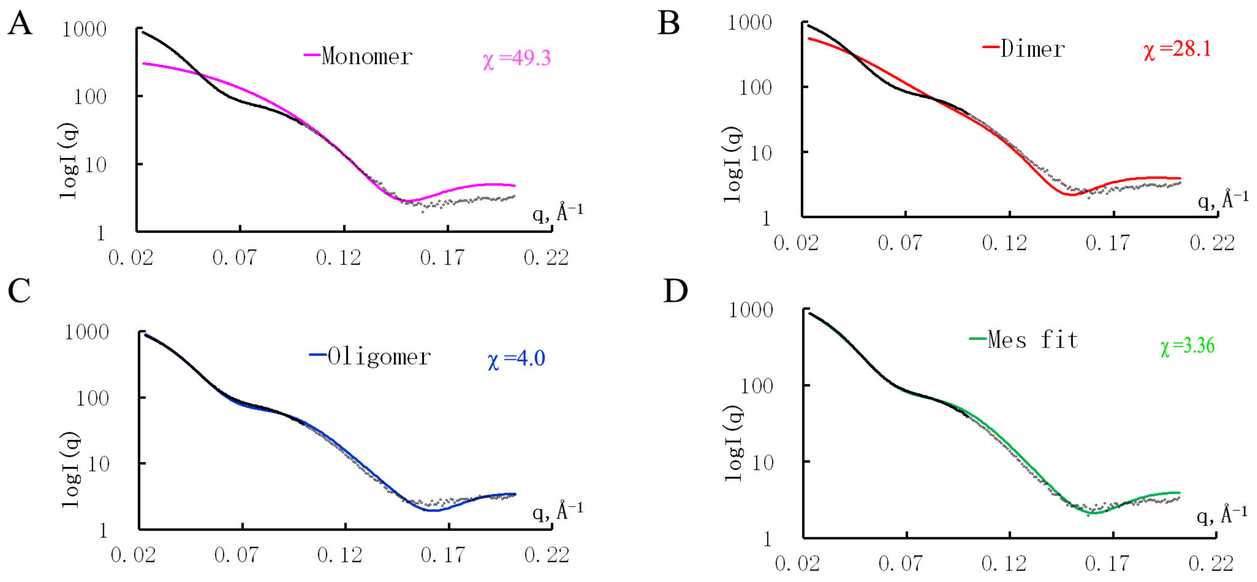

Supplementary Figure 9. The experimental and theoretical SAXS scattering curves of MRJP1 oligomer.

Experimental data are represented in black dots. The theoretical scattering curves of MRJP1 monomer (**A**, purple, extracted from solved structure), MRJP1 dimer (**B,** red, MRJP1$_2$-Apisimin$_2$-Osl$_4$), oligomer (**C,** blue, MRJP1$_4$-Apisimin$_4$-Osl$_8$) and the assembly from MES fit [7] (**D**, green) are shown. The MES approach using the above three models did not improve the fit (χ = 3.36) obviously. In the optimized mixture, the proportion of the oligomer and the dimer are 83.4% and 16.6%, respectively. Recently, the online MES server (http://modbase.compbio.ucsf.edu/foxs/index.html) only gave maximum two components in the calculation. If the dimer was not used in the calculation, the proportion of the oligomer and the monomer in the optimized mixture (χ = 3.88) are 85.0% and 15.0%, respectively. Therefore, the oligomer, the dimer and the monomer were estimated as 72.7%, 14.5% and 12.8%, respectively. The method was similar to the references [8,9], which succeeded to measure the oligomeric state in solution. Experimental data are represented in black dots at 5 mg/mL protein concentration at pH 8.0.

A

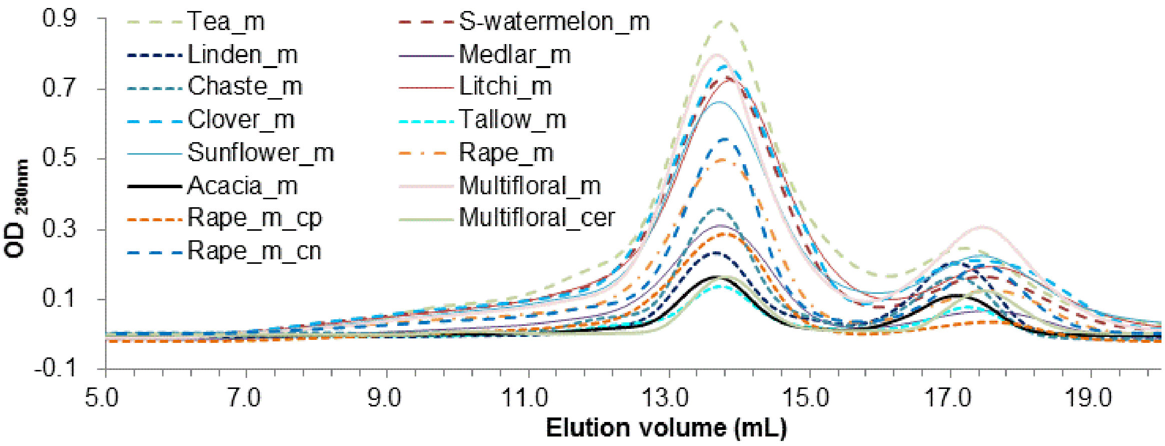

B

Name of royal jelly	Nectar plant	Honeybee species
Tea_m	Tea	*Apis mellifera*
S-watermelon_m	Seeding-watermelon	*Apis mellifera*
Linden_m	Linden	*Apis mellifera*
Medlar_m	Medlar	*Apis mellifera*
Chaste_m	Chaste	*Apis mellifera*
Litchi_m	Litchi	*Apis mellifera*
Clover_m	Clover	*Apis mellifera*
Tallow_m	Tallow	*Apis mellifera*
Sunflower_m	Sunflower	*Apis mellifera*
Rape_m	Rape	*Apis mellifera*
Acacia_m	Acacia	*Apis mellifera*
Multifloral_m	Multifloral	*Apis mellifera*
Rape_m_cn	Rape	*Apis mellifera carnica*
Rape_m_cp	Rape	*Apis mellifera carpatica*
Multifloral_cer	Multifloral	*Apis cerana*

C

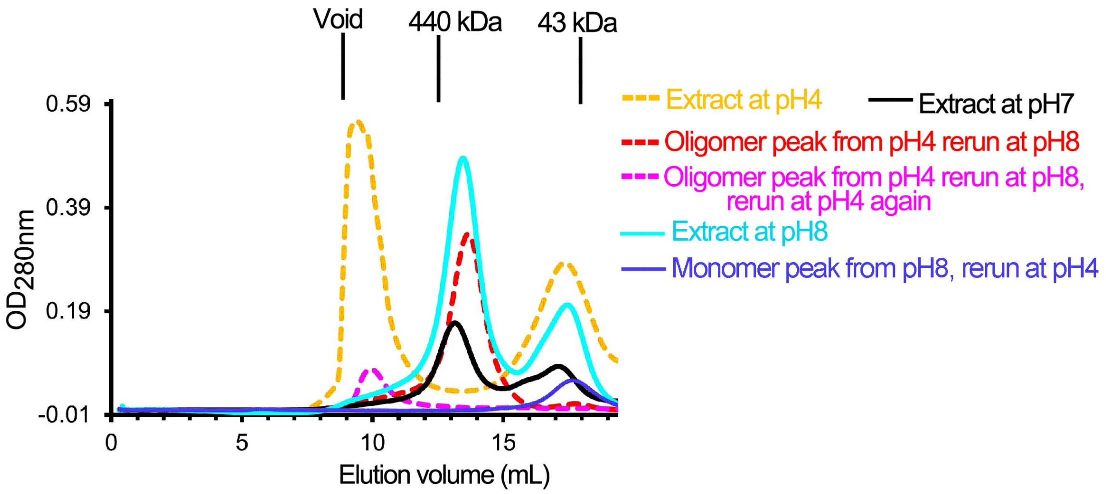

Supplementary Figure 10. MRJP1 oligomer exists universally in royal jelly and the effects of

pH on MRJP1 monomer and oligomers.

Gel filtration chromatography (**A**) and name (**B**) of royal jelly collected from nectars of different plants or honeybee species. The size exclusion chromatography of MRJP1 was performed on a Superdex 200 10/300 GL column (GE Healthcare) with the buffer containing 100 mM sodium phosphate at pH 8. (**C**) Gel filtration of MRJP1 monomer and oligomers at different pH values. MRJP1 was purified from RJ at different pH and rerun at different pH.

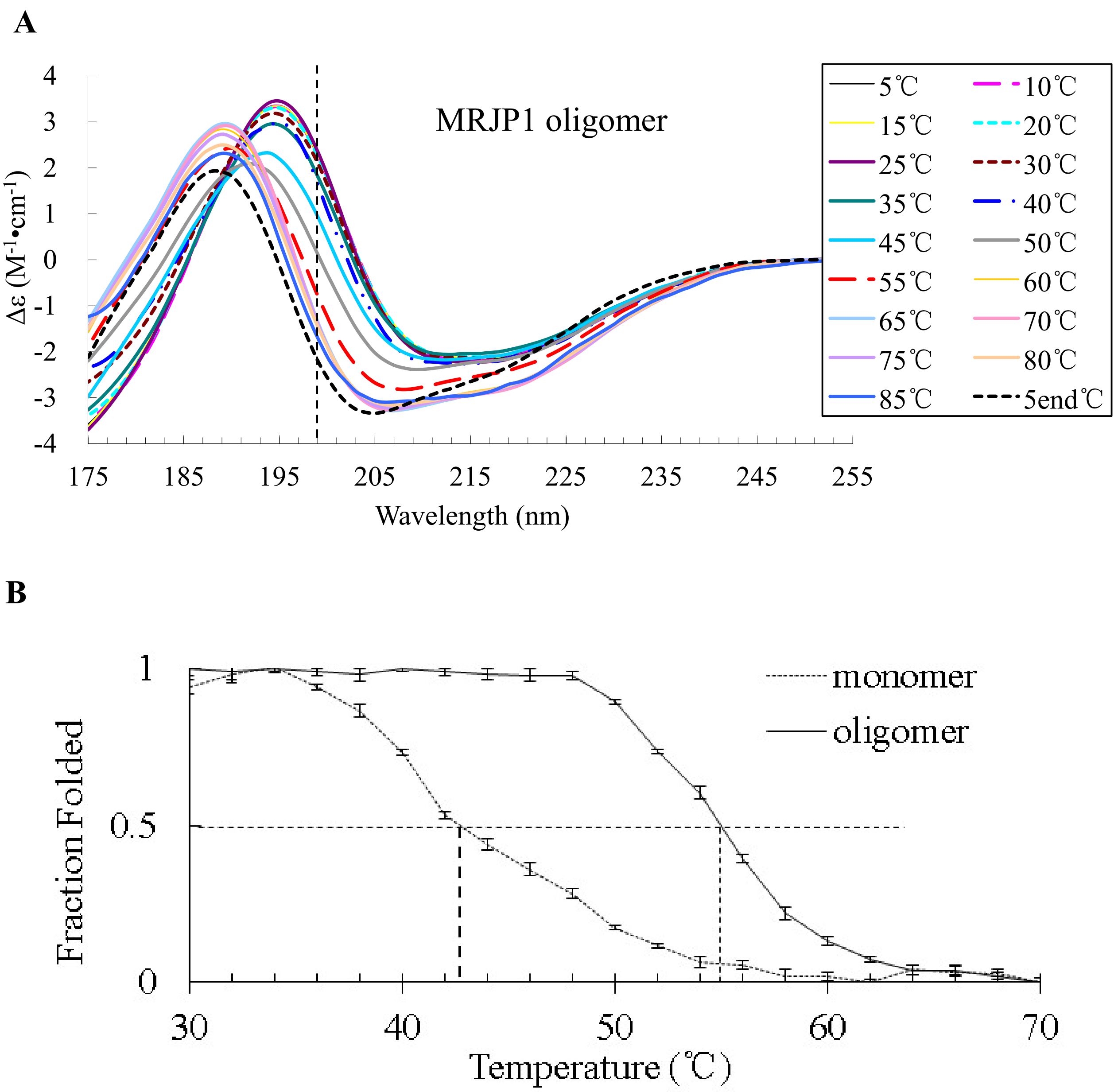

Supplementary Figure 11. Thermal denaturation curves of MRJP1 monomer and oligomer at pH8.

(**A**) The synchrotron radiation circular dichroism (CD) map of MRJP1 oligomer during the heating process. (**B**) Thermal denaturation curves of MRJP1 monomer and oligomer. Means and s.d. of fraction folded were presented from three independent experiments.

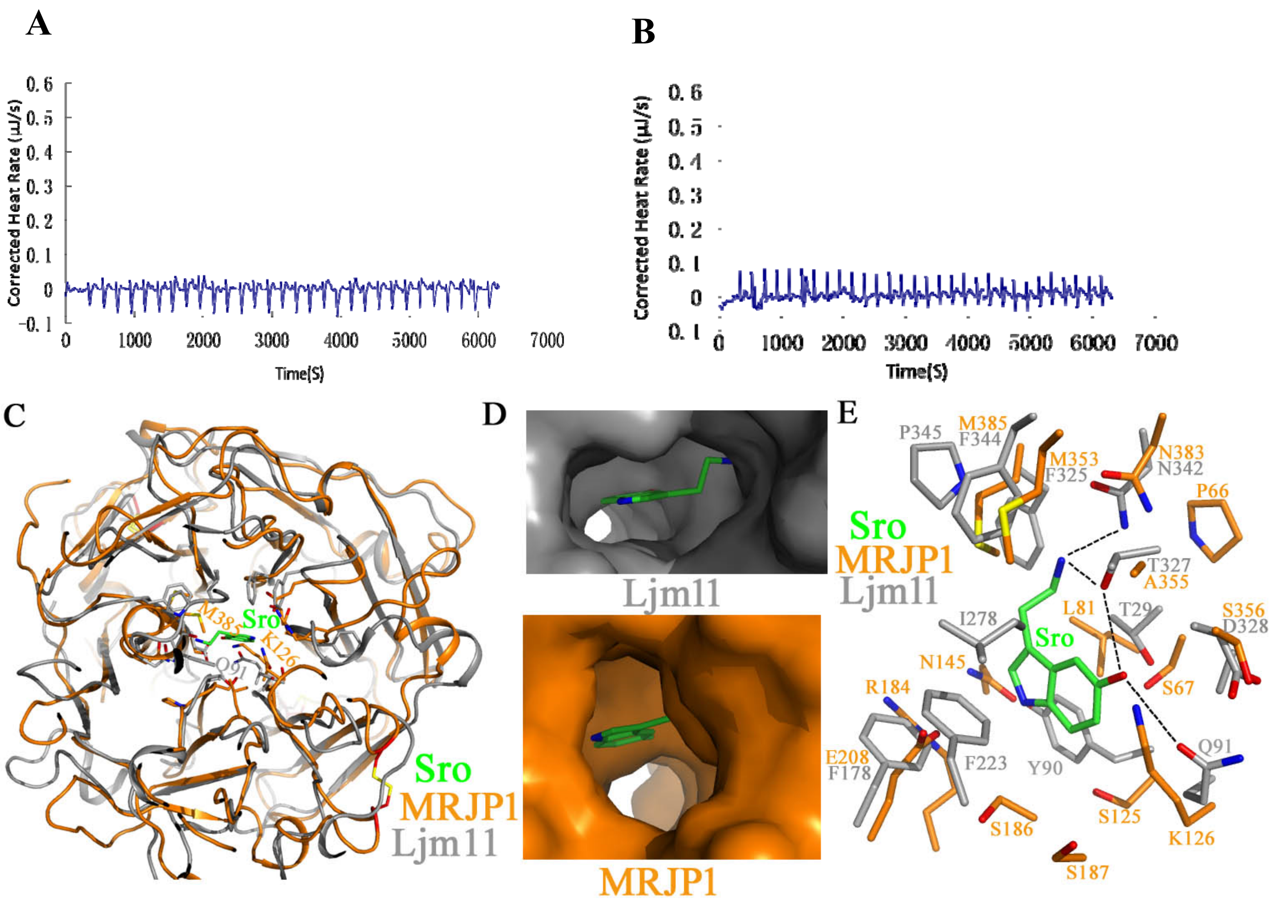

Supplementary Figure 12. Superposition of the MRJP1 structure onto Ljm11 revealed that MRJP1 might not have binding affinity to serotonin (Sro).

Quantification of the binding affinity of MRJP1 oligomer (**A**) and monomer (**B**) to serotonin by ITC. (**C**) Superposition of the MRJP1 protomer structure onto its homologous protein Ljm11 (PDB ID: 3Q6K) [10]. (**D**) The central holes of Ljm11 and MRJP1. (**E**) Residues near the holes of MRJP1 and Ljm11. Note that the position of Sro in MRJP1 was modelled from the complex structure of Ljm11. Note that the Sro was too large to be accommodated by MRJP1.

Supplementary Table 1. List of primers for MRJP1

Primer name	Sequence 5' – 3'
MRJP1 *Nde* I 1 5'	ATCTCATATGACAAGATTGTTTATGCTGG
MRJP1 *Nde* I 21 5'	ATATCTCATATGATTCTTCGAGGAGAGTCTTTAAAC
MRJP1 *Xho* I 432 S 3'	TCCTCGAGTTACAAATGGATTGAAATTTTG
MRJP1 *Nde* I 20 5'	ATATCTCATATGAACATTCTTCGAGGAGAG
MRJP1 *Nde* I 27 5'	ATATCTCATATGTTAAACAAATCATTACCCATC
MRJP1 *Nde* I 51 5'	ATATCTCATATGCAAGATGCAATTCTATCTGG
MRJP1 *Xho* I 423 S 3'	TCCTCGAGTTATCGATCATTATCGGGATT
MRJP1 *Eco*R I 20 5'	CGGAATTC AACATTCTTCGAGGAGAG
MRJP1 *Eco*R I 27 5	CGGAATTCTTAAACAAATCATTACCCATC
MRJP1 *Eco*R I 51 5'	CGGAATTCCAAGATGCAATTCTATCTGG
MRJP1 *Sal* I 423 S 3'	ACGCGTCGACTTATCGATCATTATCGGGATT
MRJP1 *Sal* I 432 S 3	ACGCGTCGACTTACAAATGGATTGAAATTTTG

Supplementary Table 2. Data-collection and scattering-fit parameters of SAXS

Data-collection parameters	
Instrument	BioSAXS station 1W2A (BSRF)
	Beamline BL19U2 (SSRF)
Wavelength (Å)	1.54
q range ($Å^{-1}$)	0.01–0.200
Exposure time	5 min for BioSAXS
	1 sec for BL19U2
Concentration range (mg ml^{-1})	0.1–10
Temperature	298 K for BioSAXS
	277 K for BL19U2
Software employed	
Data processing	FIT2D
Computation of model intensities	MES
Three-dimensional graphics representations	PyMOL

Supplementary References

1. Walter, T.S. et al. Lysine methylation as a routine rescue strategy for protein crystallization. *Structure* **14**, 1617-22 (2006).
2. Tong, L.A. & Rossmann, M.G. The locked rotation function. *Acta Crystallogr. A* **46**, 783-92 (1990).
3. Wallace, A.C., Laskowski, R.A. & Thornton, J.M. LIGPLOT: a program to generate schematic diagrams of protein-ligand interactions. *Protein Eng.* **8**, 127-34 (1995).
4. Lu, W., Zhang, C., Zeng, L. & Su, J. Synthesis of polyhydroxysterols (V): efficient and stereospecific synthesis of 24-methylene-cholest-5-ene-3beta,7alpha-diol and its C-7 epimer. *Steroids* **69**, 803-8 (2004).
5. Larkin, M.A. et al. Clustal W and Clustal X version 2.0. *Bioinformatics* **23**, 2947-8 (2007).
6. Bond, C.S. & Schuttelkopf, A.W. ALINE: a WYSIWYG protein-sequence alignment editor for publication-quality alignments. *Acta Crystallogr. D Biol. Crystallogr.* **65**, 510-2 (2009).
7. Pelikan, M., Hura, G.L. & Hammel, M. Structure and flexibility within proteins as identified through small angle X-ray scattering. *Gen. Physiol. Biophys.* **28**, 174-89 (2009).
8. Hu, Y. et al. The amino-terminal structure of human fragile X mental retardation protein obtained using precipitant-immobilized imprinted polymers. *Nat. Commun.* **6**, 6634 (2015).
9. Zhang, X. et al. Complex structures of the abscisic acid receptor PYL3/RCAR13 reveal a unique regulatory mechanism. *Structure* **20**, 780-90 (2012).
10. Xu, X. et al. Structure and function of a "yellow" protein from saliva of the sand fly Lutzomyia longipalpis that confers protective immunity against Leishmania major infection. *J. Biol. Chem.* **286**, 32383-93 (2011).
