# Supplementary material for: Architecture of the native major royal jelly protein 1 oligomer
Source: Nat Commun. 2018 Aug 22;9:3373. doi: 10.1038/s41467-018-05619-1 (PMC6105727; doi:10.1038/s41467-018-05619-1)
Supplement: Supplementary file 2 — Description of Additional Supplementary Files [file 41467_2018_5619_MOESM2_ESM.pdf]

Description of Additional Supplementary Files

File Name: Supplementary Data 1
Description: The experimental SAXS data of MRJP1 oligomer.

File Name: Supplementary Movie 1
Description: Structure of MRJP1-apisimin-24-methylenecholesterol complex and its enlargement in mp4 format.
